# High-Entropy Oxide of (BiZrMoWCeLa)O_2_ as a Novel Catalyst for Vanadium Redox Flow Batteries

**DOI:** 10.1021/acsami.3c15783

**Published:** 2024-02-20

**Authors:** Aknachew Mebreku Demeku, Daniel Manaye Kabtamu, Guan-Cheng Chen, Yun-Ting Ou, Zih-Jhong Huang, Ning-Yih Hsu, Hung-Hsien Ku, Yao-Ming Wang, Chen-Hao Wang

**Affiliations:** † Department of Materials Science and Engineering, National Taiwan University of Science and Technology, Taipei 106335, Taiwan; ‡ Department of Chemistry, Debre Berhan University, P.O. Box: 445, 000000 Debre Berhan, Ethiopia; § Chemistry Division, National Atomic Research Institute, 325207 Taoyuan, Taiwan; ∥ Maritime Innovation & Industry Promotion Department, Metal Industries Research & Development Centre, Kaohsiung 811160, Taiwan; ⊥ Hierarchical Green-Energy Materials (Hi-GEM) Research Center, National Cheng Kung University, Tainan 701401, Taiwan; # Center of Automation and Control, National Taiwan University of Science and Technology, Taipei 106335, Taiwan

**Keywords:** high-entropy oxides, vanadium redox flow battery, single phase, fluorite, graphite felt

## Abstract

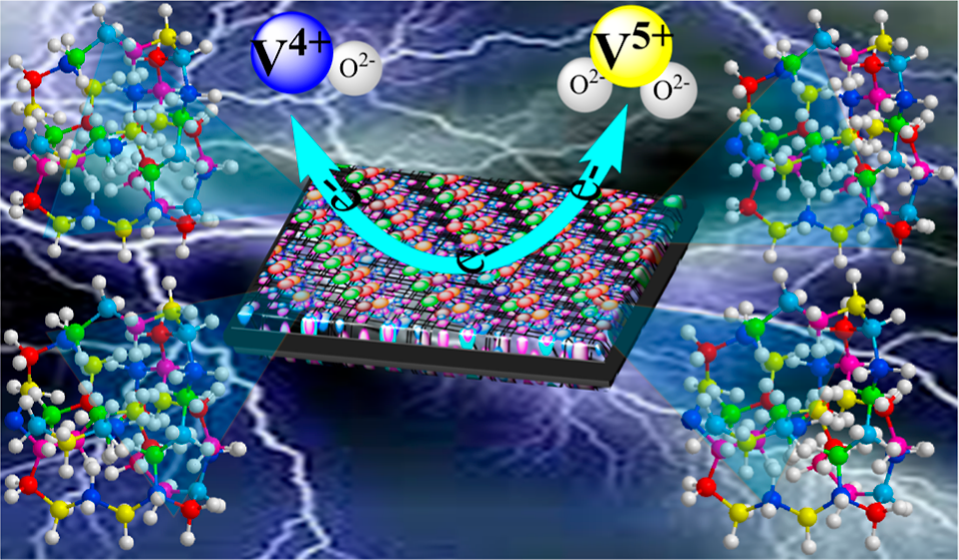

In this study, new fluorite high-entropy oxide (HEO), (BiZrMoWCeLa)O_2_, nanoparticles were produced using a surfactant-assisted hydrothermal technique followed by calcination and were used as novel catalytic materials for vanadium redox flow batteries (VRFBs). The HEO calcined at 750 °C (HEO-750) demonstrates superior electrocatalytic activity toward V^3+^/V^2+^ and VO^2+^/VO_2_^+^ redox couples compared to those of cells assembled with other samples. The charge–discharge tests further confirm that VRFBs using the HEO-750 catalyst demonstrate excellent Coulombic efficiency, voltage efficiency, and energy efficiency of 97.22, 87.47, and 85.04% at a current density of 80 mA cm^–2^ and 98.10, 74.76, and 73.34% at a higher current density of 160 mA cm^–2^, respectively. Moreover, with 500 charge–discharge cycles, there is no discernible degradation. These results are attributed to the calcination heat treatment, which induces the formation of a new single-phase fluorite structure, which facilitates the redox reactions of the vanadium redox couples. Furthermore, a high surface area, wettability, and plenty of oxygen vacancies can give more surface electroactive sites, improving the electrochemical performance, the charge transfer of the redox processes, and the stability of the VRFBs’ electrode. This is the first report on the development of fluorite structure HEO nanoparticles in VRFBs, and it opens the door to further research into other HEOs.

## 1 Introduction

The world economy is expanding rapidly, traditional nonrenewable energy sources such as coal, oil, and natural gas seem to be running out, and burning fossil fuels releases greenhouse gases such as carbon dioxide and other chemical pollutants.^1,2^ As evidence of global warming continues, scientists are trying to find green renewable energy sources, including hydroelectricity, wind energy, biomass, geothermal, and solar energy.^3^ However, even if such a transition can be made, most renewable energy sources, such as solar and wind energy, are not continuous power supplies.^4,5^ As a result, the demand for more energy storage and effective energy conversion systems must be addressed soon. Redox flow batteries (RFBs), which use liquid electrolytes to store energy while keeping electroactive species outside the battery, are a potential method for safe and additional affordable large-scale energy storage.^2,6^ This enables the system’s power and energy components to be scaled separately.^7^ Vanadium RFBs (VRFBs) are among the most affordable and efficient energy storage technologies due to their appealing qualities, such as quick reaction, high efficiency, extended cycle life, flexibility, safety, and environmentally friendly nature.^8^ Due to the use of vanadium for VRFBs during charging and discharging, cross-contamination across the ion exchange membrane is negligible.^9^ However, the comparatively high costs of production and poor energy efficiency of VRFB continue to restrict their viability despite their attractive advantages. Therefore, additional efforts must be made to improve VRFB performance and reduce manufacturing costs concerning cell layout, materials, and fabrication methods.^10^

Macroscopically, a VRFB single cell comprises an electrochemical cell with two electrolyte tanks (positive and negative), and the two electrodes are separated by an ion-exchange membrane. The positive tank holds VO_2_^+^/VO^2+^, and the negative tank holds V^2+^/V^3+^ redox couples.^11^ Theoretically, the electrolyte volume or vanadium species concentration determines the VRFB energy capacity, while the electrode or number of cells in the stack determines the VRFB’s power. The vanadium species of the positive and negative electrolytes undergo redox reactions at the electrodes during discharging, as shown in eqs 1–3.
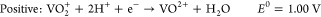
1
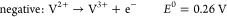
2
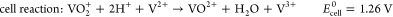
3

Overpotentials in flow cell systems are caused by concentration, charge-transfer, and ohmic polarization resistances.^8,12^ In VRFBs, the electrodes play a crucial role in system polarization, primarily due to charge-transfer polarization. Electrochemical redox reactions involving vanadium ions take place on the electrode surface, exerting a significant influence on the energy efficiency of the VRFBs. It is crucial to develop appropriate electrode and electrocatalyst materials to reduce cell overpotentials while creating the VRFBs.

Thus, graphite felt (GF) has been a popular choice for electrode material in a VRFB system due to its high anode and cathode stability, wide operating potential range, admirable conductivity, three-dimensional network structure, and affordable price.^5,13,14^ However, the use of pristine GF is hindered as an electrode material due to its poor kinetics and reversibility, limited wettability, small specific surface area, and poor electrochemical activity.^1,10,14,15^ Consequently, several electrode modification techniques, including thermal treatment, catalyst addition, and chemical treatment, have been recently developed.^2,15^ Among these, the use of catalysts to alter electrodes is a valuable way. These catalysts are classified as monometallic and metal oxides. Metallic catalysts such as Ir, Pt, and In have been used because of their high electrochemical activity, excellent conductivity, and anticorrosion properties in acidic solutions. Nevertheless, these precious metals exhibit less elemental availability and are expensive.^8^ To reduce costs, cheap metal oxides such as Nb_2_O_5_, Ta_2_O_5_, W_18_O_49_, Mn_3_O_4_, MoO_2_, PbO_2_, ZrO_2_, TiO_2_, WO_3_, and CeO_2_ have been investigated and demonstrated strong electrocatalytic activity.^13,16,17^ The increased active sites and hydrophilic metal–oxygen interaction of the metal oxide catalyze vanadium redox reactions, synergistically boosting the mass transfer rates and electron transport for vanadium redox reactions. Despite the enhanced catalysis, the adjustment of the monometallic oxide is greatly limited due to the low containment factor of the electronic structure, which quickly causes distortion and collapse of the oxide structure.^5^ Furthermore, the impact of the metal–oxygen interaction on the charge transfer of the VO^2+^/VO_2_^+^ and V^3+^/V^2+^ couples remains uncertain. Furthermore, the impact of the metal–oxygen interaction on the charge transfer of the VO^2+^/VO_2_^+^ and V^3+^/V^2+^ couples remains uncertain.

However, the task of tailoring the physicochemical properties of the materials, as mentioned earlier, without compromising their electrochemical properties remains a challenge. Therefore, designing and producing new metal oxides with enhanced electrochemical properties are imperative. The development of high-entropy ceramics (HECs) began in 2015 when Rost et al. suggested the idea of high-entropy oxides (HEOs).^18^ Recently, discovering a novel family of materials known as HEOs has attracted attention for fundamental studies and nanoengineering applications.^19^ These materials incorporate various metal cations within their crystal structure, resulting in intriguing and unexpected functional properties arising from the interactions between different cations.^20,21^ Moreover, novel synthetic techniques are employed to create this entropy-stabilized oxide system, known as HEO, which rapidly forms uncomplicated solid solution structures due to the high configurational entropy of mixing, as shown in eq 4.^18,22^ HEOs consist of at least five metallic elements, each with equal or nearly equal fractions.^23,24^ Moreover, the rise in configurational entropy causes entropy stabilization of HEOs when configuration entropies exceed 1.5*R*. This can enhance the likelihood of stabilizing a single-phase crystal structure, prompting the term “entropy-stabilized oxides” for such materials.^18,23^ In our materials, we achieved the maximum configuration entropy using the following equation. This resulted in achieving *S*_config_ = 1.75*R* and a single-phase crystal structure.
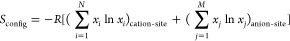
4where *x*_*i*_ denotes the mole fraction of cation sites, *x*_*j*_ represents the mole fraction of anion sites, and *R* stands for the universal gas constant.

The entropy-stabilizing effect demonstrated by HEOs provides notable benefits for electrochemical properties, including enhanced capacity and stability. These electrochemical properties of HEOs are contingent on the assortment of metal cations present, thereby enabling tuning of the electrochemical properties through elemental composition variations.^25^ The primary advantage of HEOs lies in their ability to tailor electrochemical properties via the entropy-stabilizing effect and diverse metal cations present in their crystal structure, fostering interactions that result in customized electrochemical properties. Moreover, the integration of five or more distinct cations within a single lattice structure, along with their potential synergistic effects, explains the multifunctional behavior observed.^20,26^ For instance, Sarkar et al. documented the synthesis and electrochemical charge storage properties of (Co_0.2_Cu_0.2_Mg_0.2_Ni_0.2_Zn_0.2_)O.^26^ Literature has explored functional properties of HEOs in various domains such as structural (Mg,Co,Cu,Ni,Zn)O,^27,28^ mechanical (Al_0.31_Cr_0_._20_Fe_0.14_Ni_0_._35_)O],^29^ optical [10La_2_O_3_–20TiO_2_–10Nb_2_O_5_–20WO_3_–20ZrO_2_],^30^ dielectric [(MgCoNiCuZn)O],^31^ magnetic [Ba(Fe_6_Ti_1_._2_Co_1_._2_In_1_._2_Ga_1_._2_Cr_1.2_)O_19_],^32^ supercapacitor electrodes [(CoCrFeMnNi)_3_O_4_],^33^ and physicochemical [CrFeCoNiMnOx]^34^ properties. From an application standpoint, HEOs have been investigated in diverse fields such as Li-ion batteries,^35−37^ supercapacitor electrodes,^33^ catalysis,^38,39^ and photocatalytic hydrogen production.^40^ Despite extensive studies in various areas, there has not been an attempt yet to employ pristine HEOs in the VRFBs.

This work uses a surfactant-assisted hydrothermal method to fabricate single-phase fluorite structure HEO (BiZrMoWCeLa)O_2_ nanoparticles. Our unique strategy focuses on synthesizing a HEO (Bi, Zr, Mo, W, Ce, and La) using six cations due to their favorable redox behavior and effective charge storage properties. These cations possess abundant trivalent, tetravalent, and hexavalent ions and numerous oxygen vacancies. These characteristics facilitate charge transfer during redox reactions, enhance electrode stability, create a surplus of surface electroactive sites, establish adequate pathways for electron transfer, elevate the speed of electron transport within the material, and ultimately amplify the electrochemical performance of the VRFBs. Moreover, the impact of the calcination temperature of the HEO on the electrochemical performance was explored, and we proved that HEO-750 exhibits superior catalytic performance toward V^3+^/V^2+^ and VO^2+^/VO_2_^+^ redox reactions. Hence, to create active sites and improve electrolyte accessibility, the HEO nanoparticles were decorated on the GF surface for the VO^2+^/VO_2_^+^ redox reaction, which significantly increased the hydrophilicity and electrochemical activity. As a result, the VRFB flow cell assembled with the sample of HEO calcined at 750 °C deposited on a thermally treated GF (TGF-HEO-750) electrode achieved an excellent energy efficiency of 85.04% at 80 mA cm^–2^ and 73.34% at a higher current density of 160 mA cm^–2^. These energy efficiencies were more significant than those of the VRFB cells constructed with pristine GF (PGF) and thermally treated GF (TGF). Moreover, it demonstrates remarkable cycling stability even after undergoing 500 cycles. As far as we are aware, this is the first report on developing fluorite-type HEO electrocatalysts for the VRFB application and holds significant promise for the fabrication of electrodes in the VRFBs.

## 2 Experimentation Sections

### 2.1 Chemicals/Materials

The reagents utilized in this investigation were all of analytical grade and were used exactly as supplied. GF (Ce Tech, 6.5 mm thick) was purchased. Ce(NO_3_)_3_·6H_2_O (AT J.T. Baker, 99.5%), Bi(NO_3_)_3_·5H_2_O (Alfa Aesar, 95%), La(NO_3_)_3_·6H_2_O (J.T. Baker, 99%), Na_2_ MoO_4_·2H_2_O (USA Alfa Aesar, 99%), Na_2_ WO_4_·2H_2_O (India Alfa Aesar, 98+%), ZrOCl_2_·8H_2_O (China Alfa Aesar, 98.5%), CTAB (India Alfa Aesar, 99%), urea, and ethanol were purchased. Deionized (DI) water was procured from Millipore.

### 2.2 Powder HEO Synthesis

**Scheme 1:**
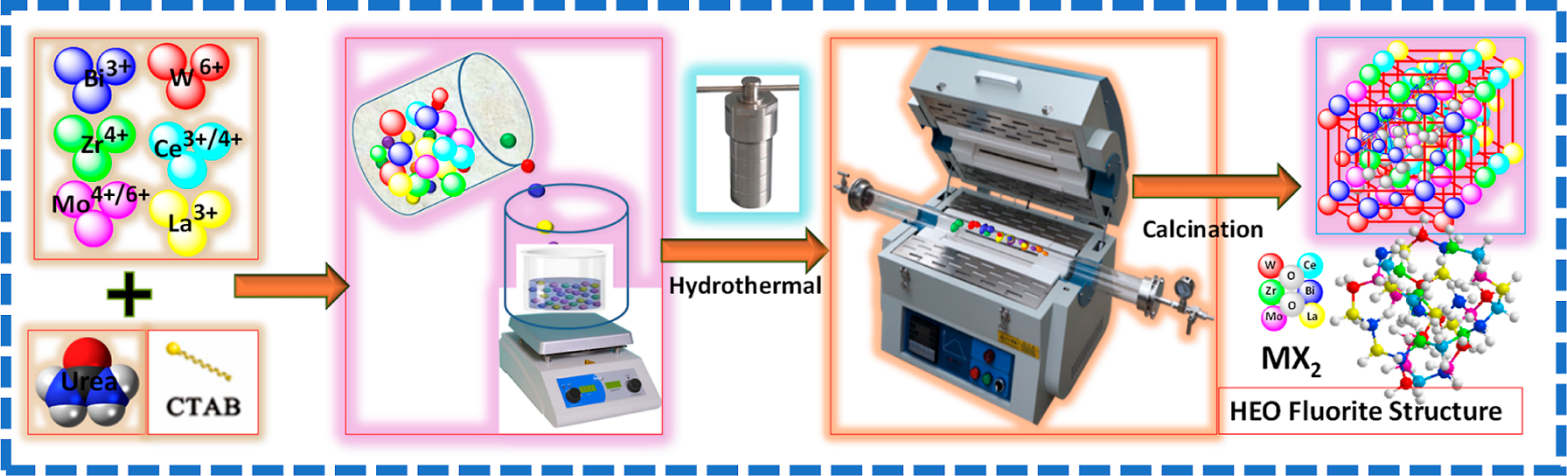
Schematic Diagram for the Preparation of HEOs

The HEOs were synthesized using a hydrothermal technique by adding a surfactant, in which the precursor solution was made by mixing Na_2_MoO_4_·2H_2_O (0.2 mmol), Bi(NO_3_)_3_·5H_2_O (0.2 mmol), La(NO_3_)_3_·6H_2_O (0.1 mmol), Ce(NO_3_)_3_·6H_2_O (0.1 mmol), Na_2_ WO_4_·2H_2_O (0.2 mmol), ZrOCl_2_·8H_2_O (0.2 mmol), and CTAB (0.625 mmol) in 40.0 mL of DI water.^35^ When the solution was clear, urea was added. The urea (1.2 mmol) molar ratio to all metal salts was 6:1. Subsequently, it was moved to hydrothermal treatment at 180 °C for 12, 18, and 24 h. The mixtures were separated by centrifugation followed by rinsing with ethanol and DI water and dried for 12 h in a vacuum oven. The powdered sample was then heated for 2 h in air at 550, 750, and 900 °C with a ramp of 10 °C min^–1^, denoted by HEO-550, HEO-750, and HEO-900, respectively. The general synthetic procedure for fluorite structure HEO (BiZrMoWCeLa)O_2_ nanoparticles is illustrated in Scheme 1.

### 2.3 Physicochemical, Morphological, and Structural Characterization

A field emission scanning electron microscope was used to examine the morphology (JSM-6500F). An X-ray field emission gun transmission electron microscope (FEI Tecnai G2 F-20 S-TWIN) was used to obtain the morphology and *d*-spacing. X-ray diffraction (XRD) (Bruker, D2-PHASER X-ray Diffractometer) was used to analyze the phases and crystallographic structures. A Raman spectrometer (iHR550) with a 532 nm laser beam detected the molecules’ vibration states to discover the materials’ bonding conditions and structural flaws. Fourier transform infrared (FT-IR, JASCO FT/IR6200) was applied to identify the unknown chemical composition of the materials. X-ray photoelectron spectroscopy (XPS, Thermo, K-Alpha) was used to assess the binding energy between the surface atoms of materials. Brunauer–Emmett–Teller (BET, NOVA touch LX^2^) analysis was performed to determine the materials’ specific surface area and porosity. Contact angle testing (FTA-125) was used to determine the wetting characteristics. UV–vis spectroscopy (V-670) was utilized for the dissolution test.

### 2.4 Electrochemical Measurements

Cyclic voltammetry (CV) was measured using a standard three-electrode setup and an electrochemical workstation (Bio-Logic, VSP-300). A glassy carbon-ring disk electrode (RDE, 0.196 cm^2^) was used as the working electrode (WE), Ag/AgCl as the reference electrode (RE), and platinum wire as the counter electrode (CE). The RDE ink contained 7.5 mg catalyst, IPA (2.8 mL), DI water (2.8 mL), and 5 wt % Nafion solution (0.04 mL). The electrolyte comprised 1.6 M VOSO_4_ and 4.6 M H_2_SO_4_, with positive and negative scan ranges from 0.0 to 1.5 V and from −1.0 to 0.0 V, respectively, at 10 mV s^–1^. To minimize the undesired oxidation of the active species, the electrolyte was purged with N_2_. Furthermore, linear sweep voltammetry (LSV) experiments were conducted with rotating speeds from 200 to 2000 rpm and a scan rate of 0.002 V s^–1^ using the same electrolyte as that employed in RDE CV measurements. Electrochemical impedance spectroscopy (EIS) test was performed with an applied potential of 1.0 V and a frequency range of 100 kHz–10 mHz. For GF electrode CV tests, GFs with an area of 1.327 cm^2^ and 6.5 mm thickness were used with an electrolyte of 0.05 M VOSO_4_ in 2 M H_2_SO_4_ and a scan rate of 5 mV s^–1^. EIS was conducted at an AC voltage of 10 mV and frequencies from 100 kHz to 10 mHz. In situ Raman spectra were obtained using a UniDRON system with a 532 nm laser, spanning potentials from 0.6 to 1.6, which were controlled by an Autolab PGSTAT 204 workstation.

### 2.5 Incorporation of Catalyst on GF

**Scheme 2:**
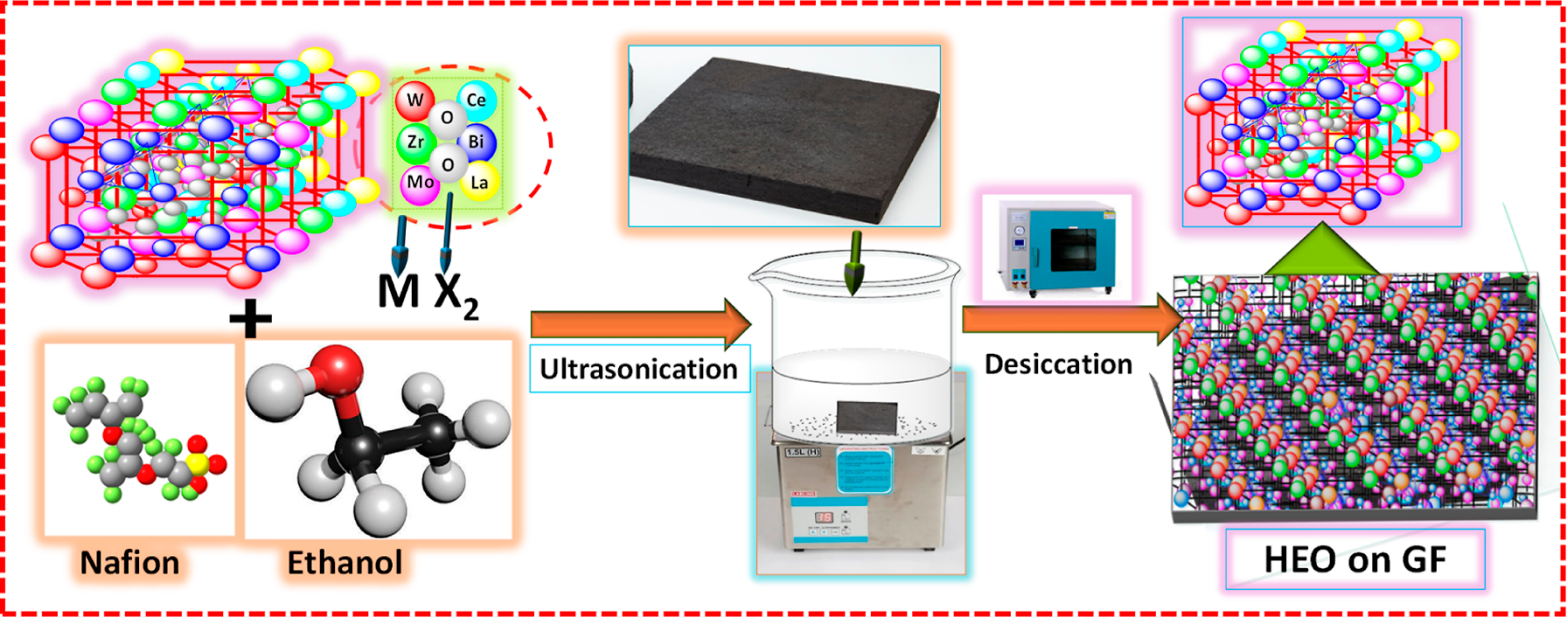
Schematic Diagram for the Preparation of HEOs on GF

The catalyst-loaded GF was prepared for an electrochemical performance test. The sample of HEO calcined at 750 °C was deposited on a thermally treated GF (TGF-HEO-750) electrode made by mixing 25 mg of the HEO-750 with 5 wt % Nafion (5 mL) and ethanol (40 mL), then ultrasonicated for 1 h. Subsequently, the thermal-treated GF was immersed in ink and subjected to ultrasonication for 5 min. Afterward, the treated GF was placed at 80 °C for 30 min. This step was continued until the entire electrolyte was fully utilized. Once the ink-drying process was completed, the catalyst-loaded GF was allowed to dry at 80 °C for 24 h. The general synthetic procedure for incorporating catalyst on GF is illustrated in Scheme 2.

### 2.6 Single-Cell Tests

The battery tests of the VRFB were conducted using a solution containing 1.6 M VOSO_4_ plus 4.6 M H_2_SO_4_. The positive electrode was equipped with GFs (5 × 5 × 0.65 cm) containing the TGF-HEO-750, while the negative electrode utilized TGF. The Nafion 212 membrane (DuPont) was sandwiched between the cell stacks. Both electrolyte bottles contain 60 mL and use two FMI pumps at a flow rate of 80 mL min^–1^. The charge–discharge tests were conducted at a potential window of 0.7–1.6 V, employing numerous current densities (80, 100, 120, 140, and 160 mA cm^–2^) to assess the Coulombic efficiency (CE), energy efficiency (EE), and voltage efficiency (VE) using eqs 5–7. Additionally, stability tests were performed to assess the operational long-term viability of different electrodes, pristine GF (PGF), TGF, and TGF-HEO-750 after 100 cycles at 120 mA cm^–2^. Notably, the TGF-HEO-750 electrodes exhibited exceptional stability, sustaining 500 cycles at 120 mA cm^–2^.
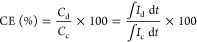
5
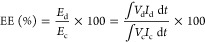
6
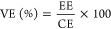
7where *C*_d_: discharge capacity, *C*_c_: charge capacity, *I*_d_: discharge current, *I*_c_: charge current, *E*_d_: discharge energy, *E*_c_: charge energy, *V*_d_: discharge voltage, and *V*_c_: charge voltage.

## 3 Results and Discussion

**Figure 1:**
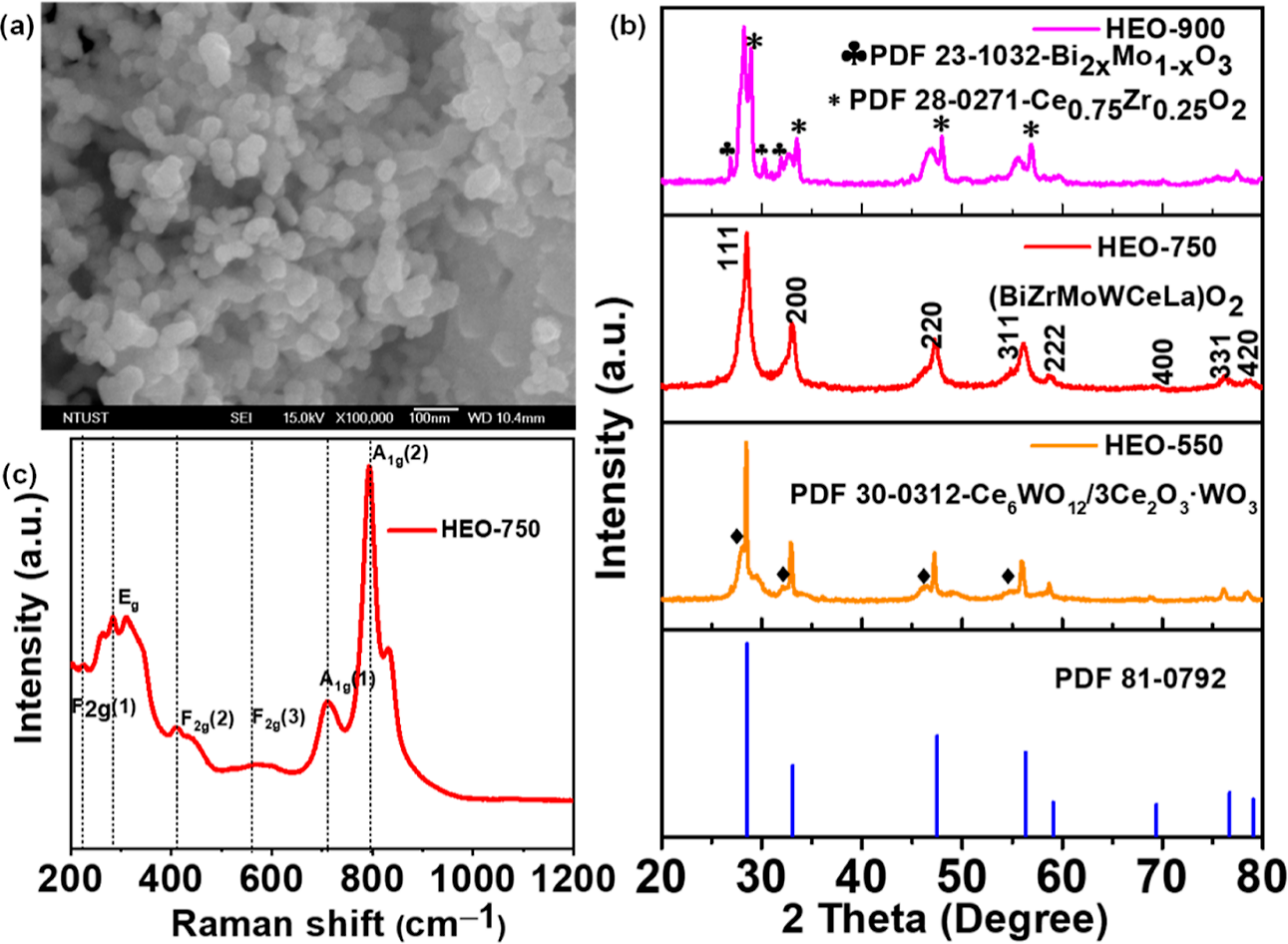
(a) SEM image of HEO-750, (b) XRD pattern of HEO-550 (orange), HEO-750 (red), and HEO-900 (pink), and (c) Raman spectrum of HEO-750 nanoparticles.

The surface morphology and characteristics of HEO are shown in Figures 1 and S1. The calcination temperature at 550 °C includes submicrometer-sized aggregates of nanoparticles, as shown in Figure S1a. The aggregates separate by raising the calcination temperature to 750 °C, as shown in Figures 1a and S1b. It shows that all metallic elements have a uniform size distribution, confirming the presence of chemical and microstructural homogeneity.^35^Figure S1c illustrates the aggregations of nanosized particles at 900 °C. The pore size in the Brunauer–Emmett–Teller (BET) analysis of nanoparticles slightly collapses with increasing calcination temperature.^41,42^Figure S2 displays the BET-specific surface area and the inset presenting the pore size for HEO-750 and HEO-900 samples. The isotherms reveal mesoporous structures characterized by pore sizes ranging from 2 to 50 nm.^38,43^ The calculated BET-specific surface areas of HEO-750 and HEO-900 are 54.74 and 13.12 m^2^ g^–1^, respectively. Table S1 contains the calculated values for the pore volume and diameter for HEO-750 and HEO-900. The energy-dispersive spectrometry (EDS) elemental compositions of the three samples, HEO-550, HEO-750, and HEO-900, are shown in Figure S3, demonstrating the presence of each element in each sample.

The XRD pattern was acquired to validate the structure and phase scanned within the 2θ range 20–80°, as shown in Figures 1b and S4b. The distinct metal cations cannot yet form a single-phase crystal structure at low calcination temperatures;^44,45^ intermediate products were prepared at 550 °C [Figure 1b (orange)]. The sample prepared at 750 °C demonstrates well-defined diffraction peaks for the HEO nanostructure and single-phase fluorite crystal structure formation, as observed in Figure 1b (red). The XRD peaks observed at specific angles (2θ = 28.44, 33.12, 47.38, 56.24, 59.09, 69.31, 76.69, and 79.16°) correspond to crystallographic planes: (111), (200), (220), (311), (222), (400), (331), and (420), respectively, as per the (PDF card #81-0792). These peaks align perfectly with the bulk XRD pattern, confirming a single-phase cubic [face-centered cubic (fcc)] HEO fluorite structure, consistent with the space group (*Fm*3̅*m*, 225, *a* = 5.41).^44,46^ However, at an increased calcination temperature of 900 °C [Figure 1b (pink)], alterations in the diffraction peaks within the XRD pattern signify a notable phase transition.^20^ The significant difference (δ) in the radius of the cation of site A, which leads to the formation of the second phase, is accountable for the excessive lattice distortion energy.^47^Figure S4b shows further investigation of the samples obtained at a calcination temperature of 750 °C for various hydrothermal treatments showing distinct crystalline phases. The Raman spectrum confirms the presence of six phonon modes on the symmetry assignment (Figure 1c). The Raman active modes of 2A_1g_(1,2), F_2g_(3), F_2g_(2), F_2g_(1), and E_g_, respectively, are responsible for the peaks, which are located in the ranges of 610–730, 560–610, 470–560, 210, and 290–430 cm^–1^, respectively. These modes of vibration correspond to the tetrahedral symmetry of the metal–oxygen bonds.^38,48−50^ A system with six cations of various ionic sizes that occupied random sites is expected to have a high-intensity mode at ∼800 cm^–1^. The presence of the F_2g_ band and broadening are common characteristics frequently linked to chemical substitutions, which cause crystal lattices to expand, M–O bond lengths to vary, optical phonon confinement to exist in nanomaterials, and the formation of oxygen defects.^38,49^Figure S4a shows the FTIR spectrum of HEO-750. The bands observed within the range of 452–608 cm^–1^ are attributed to the bonds formed between the metal and oxygen.^49^ In summary, both the Raman and FTIR studies confirm the formation of the fluorite structure of HEO (BiZrMoWCeLa)O_2_ nanoparticles.

**Figure 2:**
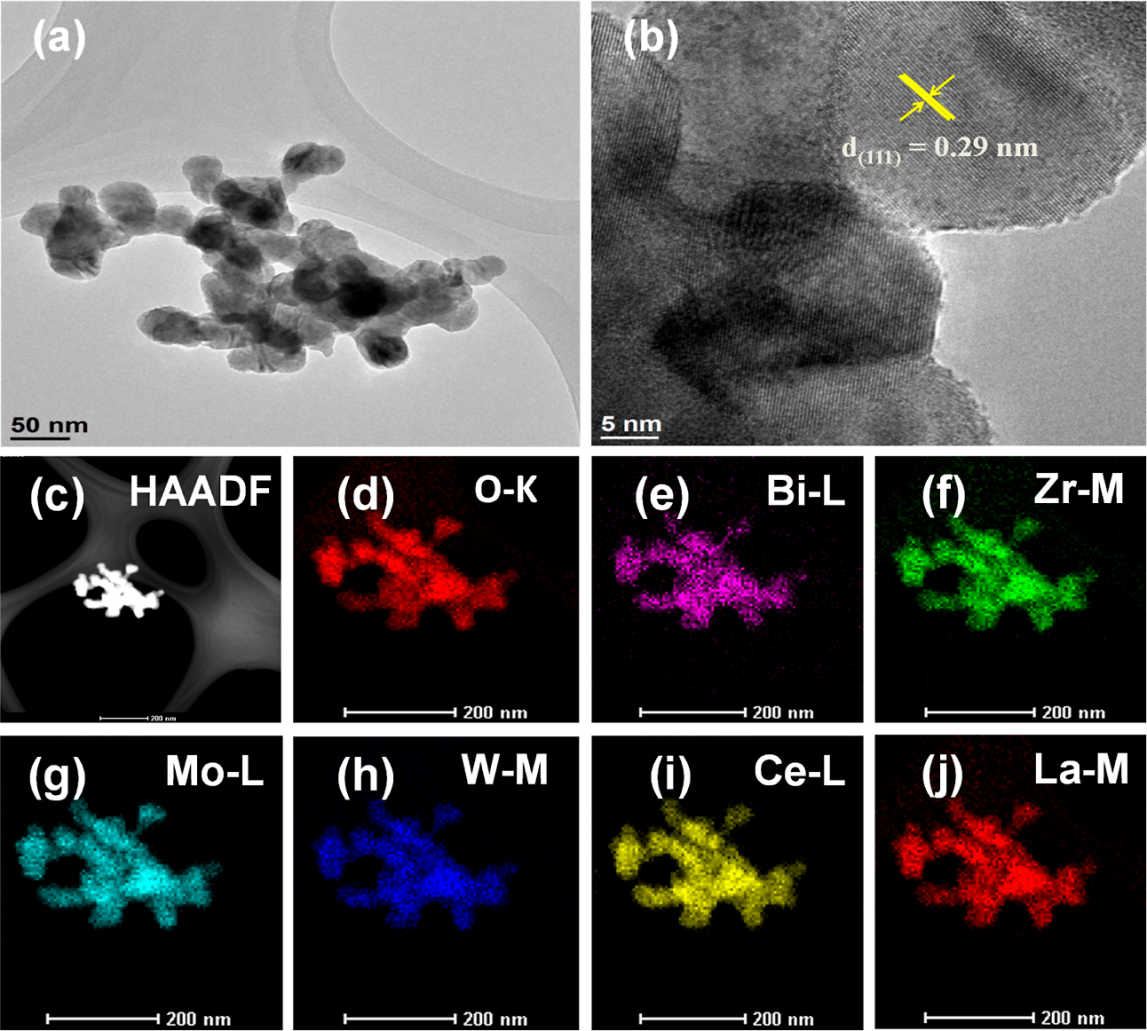
(a) TEM image, (b) HR-TEM image, (c) HAADF-STEM, and (d–j) mapping of the HEO-750 nanoparticles.

The morphology and structure were further investigated using transmission electron microscopy (TEM) analysis, which revealed the uniform distribution of the HEO nanoparticles, as depicted in Figure 2a. The high-resolution transmission image of HEO-750 (Figure 2b) shows the fcc crystal structure plane (111) of *d*-spacing (0.29 nm) and matches the XRD findings. These results confirmed the formation of HEO (BiZrMoWCeLa)O_2_ fluorite structure nanoparticles. The high-angle annular dark-field scanning transmission electron microscopy (HAADF-STEM) image of the HEO-750 nanoparticles is shown in Figure 2c, and elemental mapping images are shown in Figure 2d–j. The sample region shows a distinct compositional profile of the HEO, with Bi, Zr, Mo, W, Ce, La, and O elements present in a uniform manner. In short, it suggests all cation species’ homogeneous chemical distribution and microstructural homogeneity.^39,49^

**Figure 3:**
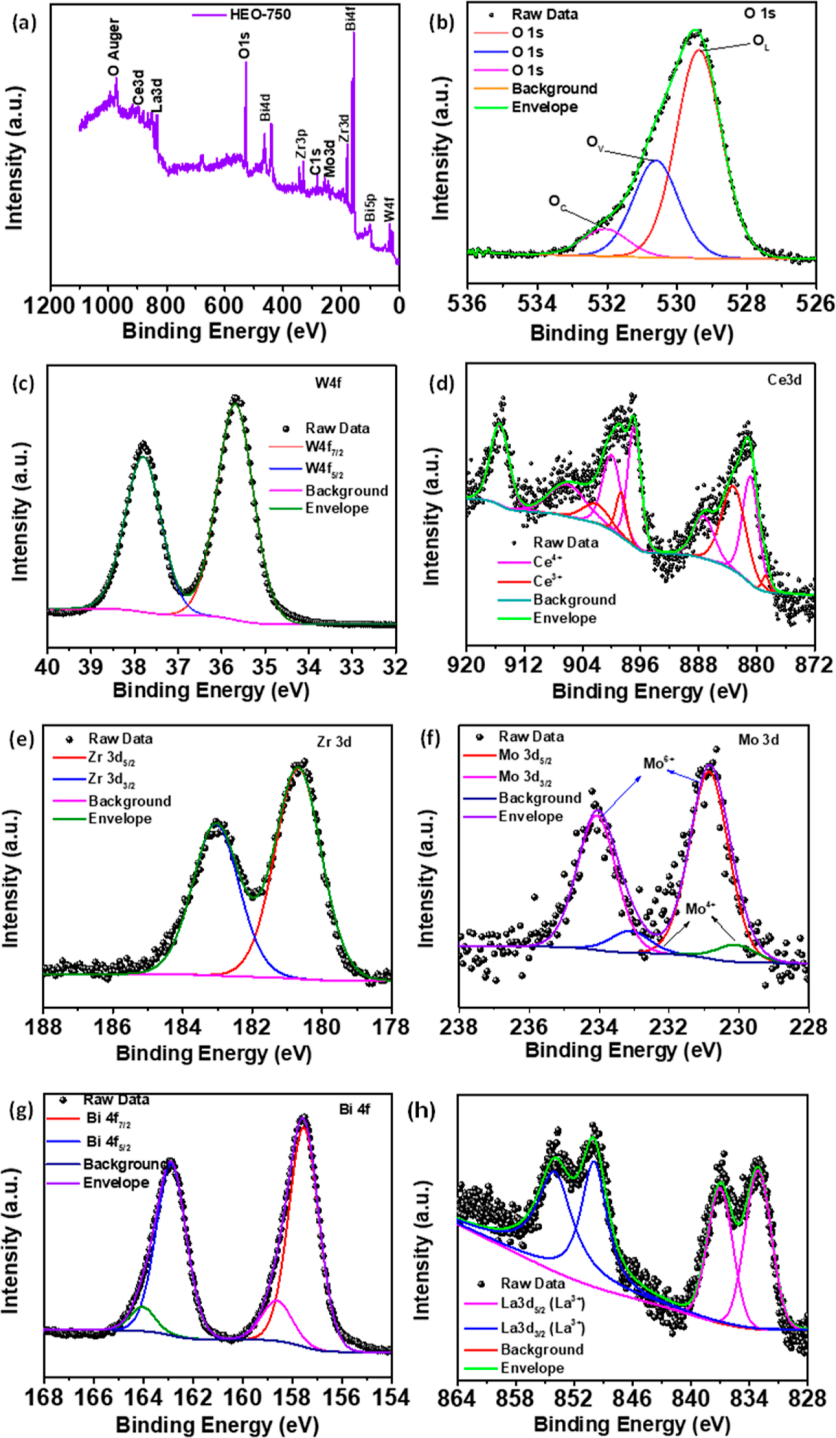
XPS spectra of the HEO-750: (a) element survey, (b) O 1s, (c) W 4f, (d) Ce 3d5, (e) Zr 3d, (f) Mo 3d, (g) Bi 4f, and (h) La 3d5.

The XPS spectra (Figure 3) investigated the elemental composition and oxidation states of HEO-750. Figure 3a confirms the signals for La, Bi, Ce, Zr, Mo, W, and O. The O 1s spectrum revealed three peaks at 529.3 eV (O_L_), 530.6 eV (O_V_), and 532 eV (O_C_) (Figures 3b and S5b), indicating lattice oxygen, oxygen vacancies, and surface-adsorbed oxygen species, respectively.^49^ The presence of significant oxygen vacancies (Ov) was indicated by peak area ratios, contributing significantly to the O 1s spectrum. These vacancies act as electroactive centers, enhancing electrical conductivity and specific capacitance during rapid surface redox processes.^51^ Moreover, Figure S5 displays the XPS spectra of O 1s for HEO-550, HEO-750, and HEO-900. Table S2 presents the corresponding percentages obtained from fitting the O 1s spectra. As depicted in Figure 3c, the W 4f spectrum exhibits prominent peaks at energies of 37.8 and 35.6 eV, which correspond to the binding energies of electrons in the W 4f_5/2_ and W 4f_7/2_ orbitals of tungsten in the W^6+^ oxidation state, respectively.^52^ Ce 3d doublets (Ce 3d_5/2_ and Ce 3d_3/2_) presented in the sample can be associated with Ce^3+^ and Ce^4+^ oxidation states, respectively, with different binding energies, as shown in Figure 3d. Detailed spectra, which provide details on the binding energies of Ce^4+^ and Ce^3+^, can be found in the results published in the literature.^48,53,54^Figure 3e shows the Zr 3d spectrum binding energy values of 180.0 0 eV (Zr 3d_5/2_) and 183 eV (Zr 3d_3/2_), indicating emissions from Zr^4+^ sites.^55^ The Mo 3d spectrum exhibits two prominent peaks at 230.8 eV (Mo 3d_5/2_) and 234 eV (Mo 3d_3/2_) (Figure 3f) and are mainly Mo^6+^ with a small amount of Mo^4+^.^56,57^ In Figure 3g, the Bi 4f spectrum shows two distinct peaks at 157.5 and 162.8 eV (4f_7/2_ and Bi 4f_5/2_, respectively), indicating the presence of Bi^3+^ in Bi_2_O_3_. Additionally, two less prominent peaks may arise from a small effect of surface charging induced by the crystal’s polarization shift. Satellite peaks with a distance of approximately 5.3 eV from the prominent peaks of Bi 4f_7/2_ and Bi 4f_5/2_ are consistent with the reported values.^58^Figure 3h exhibits the La 3d spectra, two doublets (La 3d_5/2_ and La 3d_3/2_), binding energies of 833.17 and 837.04 eV, and peak of 849.93 and 854.11 eV, respectively. The binding energy indicates the presence of La^3+^ and agrees with the reported literature results.^48^ Generally, XPS investigations verified the presence of all cations within the HEO-750 sample.

**Figure 4:**
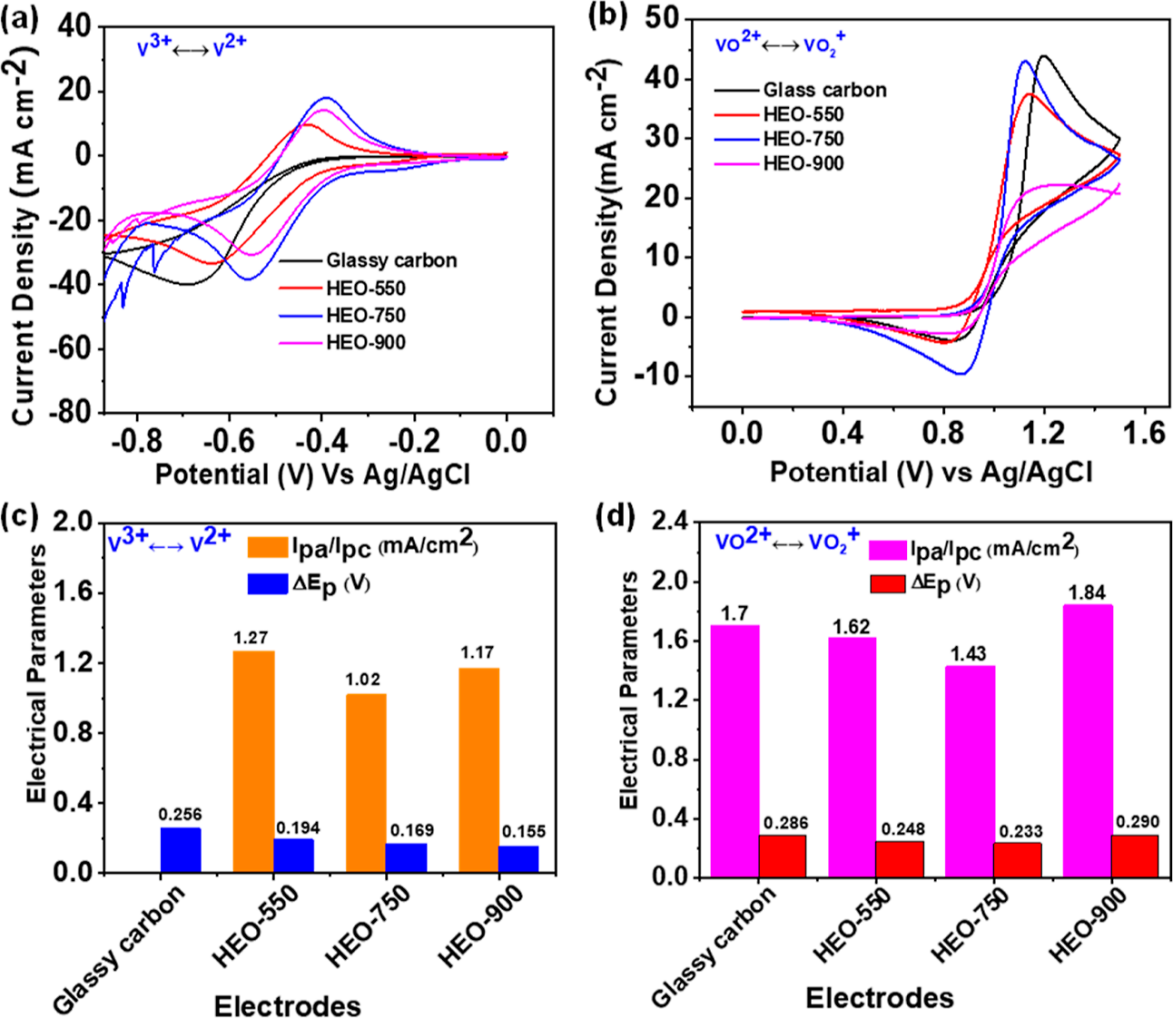
CV curves of the HEO (550, 750, and 900) and estimated values of *I*_pa_/*I*_pc_ and Δ*E*_p_, respectively, in (a,c) low potential range and (b,d) high potential range in the electrolyte of 1.6 M VOSO_4_ + 4.6 M H_2_SO_4_ at 10 mV s^–1^.

CV tests were performed to assess the catalytic activity of the glassy carbon, HEO-550, HEO-750, and HEO-900 catalysts toward vanadium redox reactions in 1.6 M VOSO_4_ + 4.6 M H_2_SO_4_ at 10 mV s^–1^. In Figure 4a,b, HEO-750 indicates the highest peak current densities, indicating superior electron mobility toward V^2+^/V^3+^ and VO^2+^/VO_2_^+^ redox reactions. Figure S17b shows the CV curves of HEO-750 repetitive cycles of 5th, 15th, 30th, 50th, 80th, 100th, 150th, and 200th in the electrolyte solution of 1.6 M VOSO_4_ + 4.6 M H_2_SO_4_. The smaller peak potential separations (Δ*E*_p_) of HEO-750 among all samples are displayed in Figure 4c,d. This suggests that the HEO-750 sample has better electrochemical reversibility toward both redox couples than those of the other samples. Furthermore, the peak current density ratios for the HEO-750 sample are close to the ideal values of 1.43 and 1.02 mA cm^–2^ for the positive and negative electrodes, respectively. The above results indicate that HEO-750 has comprehensive electrochemical activity due to its active sites for both electrode systems. Unfortunately, when the HEO-750 catalyst is used on the negative side, the hydrogen evolution reaction (HER) side reaction is more prone. Due to this reason, the HEO catalyst was used on the positive side only during battery tests. However, the peak separations of HEO-900 increase significantly in addition to the disappearance of the reduction peak in the positive electrode, indicating an insufficient catalytic activity. Thus, the optimal electrocatalytic activity of HEO reaches 750 °C. Additional CV data obtained at different hydrothermal treatment temperatures and reaction times on the electrocatalytic activity of HEO for both negative and positive electrodes are available in Figure S6. Additionally, the current density ratios (*I*_pa_/*I*_pc_) and potential separation (Δ*E*_p_) of different electrode systems are listed in Table S3. The distinct redox peaks of CV curves are due to fast reactions involving the oxidation states of Bi^3+^/Bi^0^, La^3+^/La^0^, Ce^3+/4+^/Ce^0^, Zr^4+^/Zr^0^, Mo^4+/6+^/Mo^0^, and W^6+^/W^0^.^43,58^ Additionally, the CV curves for VO^2+^/VO_2_^+^ on the HEO-750 electrode were performed at varying scan rates, displaying peak currents (*i*_pa_ and *i*_pc_) in Figure S7a,b. The electrode reaction rates of HEO-750 were assessed through linear sweep voltammetry (LSV) at different rotation speeds of the i_L_ and the relationship between the ω^1/2^, as shown in Figure S8a,b, respectively. Figure S8c is obtained by taking the logarithm of the reciprocal of the y-intercepts in Figure S8d. Furthermore, the potential dependent in situ Raman spectra of HEO-750 in 0.05 M VOSO_4_and 2 M H_2_SO_4_ at various potentials are shown in Figure S9a–d. Figure S9a,b displays spectra with low wavenumbers, while Figure S9c,d displays spectra with high wavenumbers.

**Figure 5:**
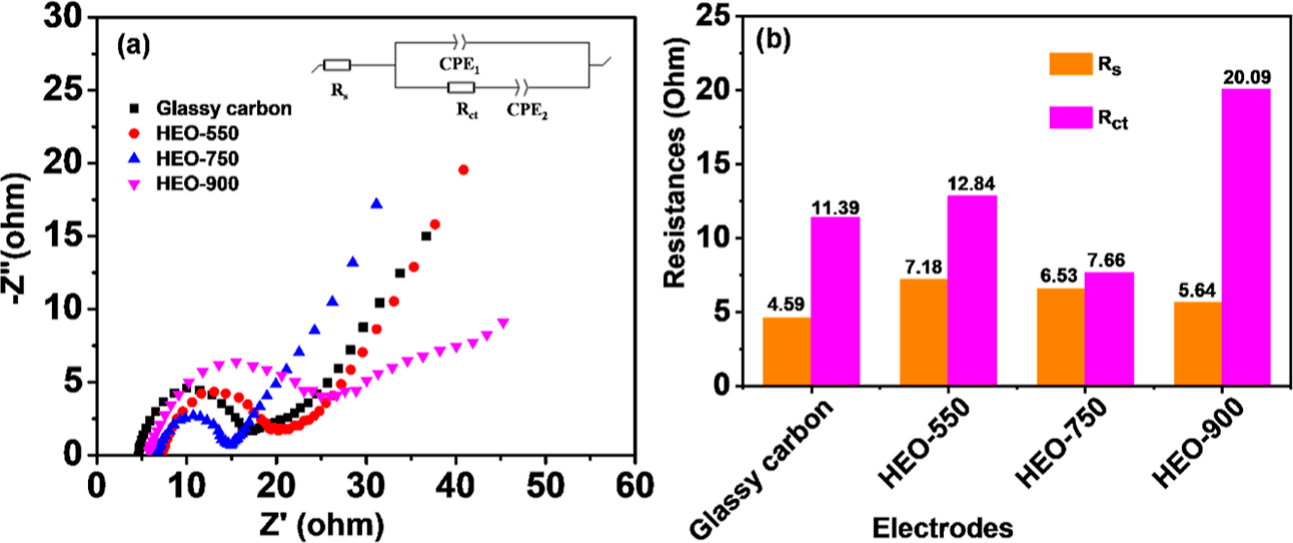
(a) EIS curves of the HEO-(550, 750, and 900) electrodes, and (b) estimated values obtained from the curves in the electrolyte of 1.6 M VOSO_4_ + 4.6 M H_2_SO_4_ at a polarization potential of 1.0 V.

Electrochemical impedance spectroscopy (EIS) was utilized to assess electron transfer and electrode catalytic activity. Nyquist plots revealed a high-frequency semicircle and a low-frequency sloping line, suggesting a mixed-controlled process involving charge transfer and diffusion steps.^59^ The plots for catalysts HEO-550, HEO-750, and HEO-900 at a potential of 1.0 V are given in Figure 5. Additionally, Nyquist plots for various HEO samples under different hydrothermal conditions are provided in Figure S10. *R*_s_ represents solution resistance, with lower values indicating better electrolyte ionic conductivity. The semicircle diameter determines the charge transfer resistance (*R*_ct_), with lower *R*_ct_ values indicating a stronger interaction of electrolytic ions with the electrode material. Nyquist plots were fitted using a circuit diagram (Figure 5a inset), and the results are in Table S4 and Figure 5b. In the corresponding circuit, CPE denotes the constant phase element compensating for the solution/electrode interface’s double-layer capacitance. Therefore, the HEO-750 catalyst has the lowest *R*_ct_ values, indicating a superior electrochemical activity than that of all samples.

**Figure 6:**
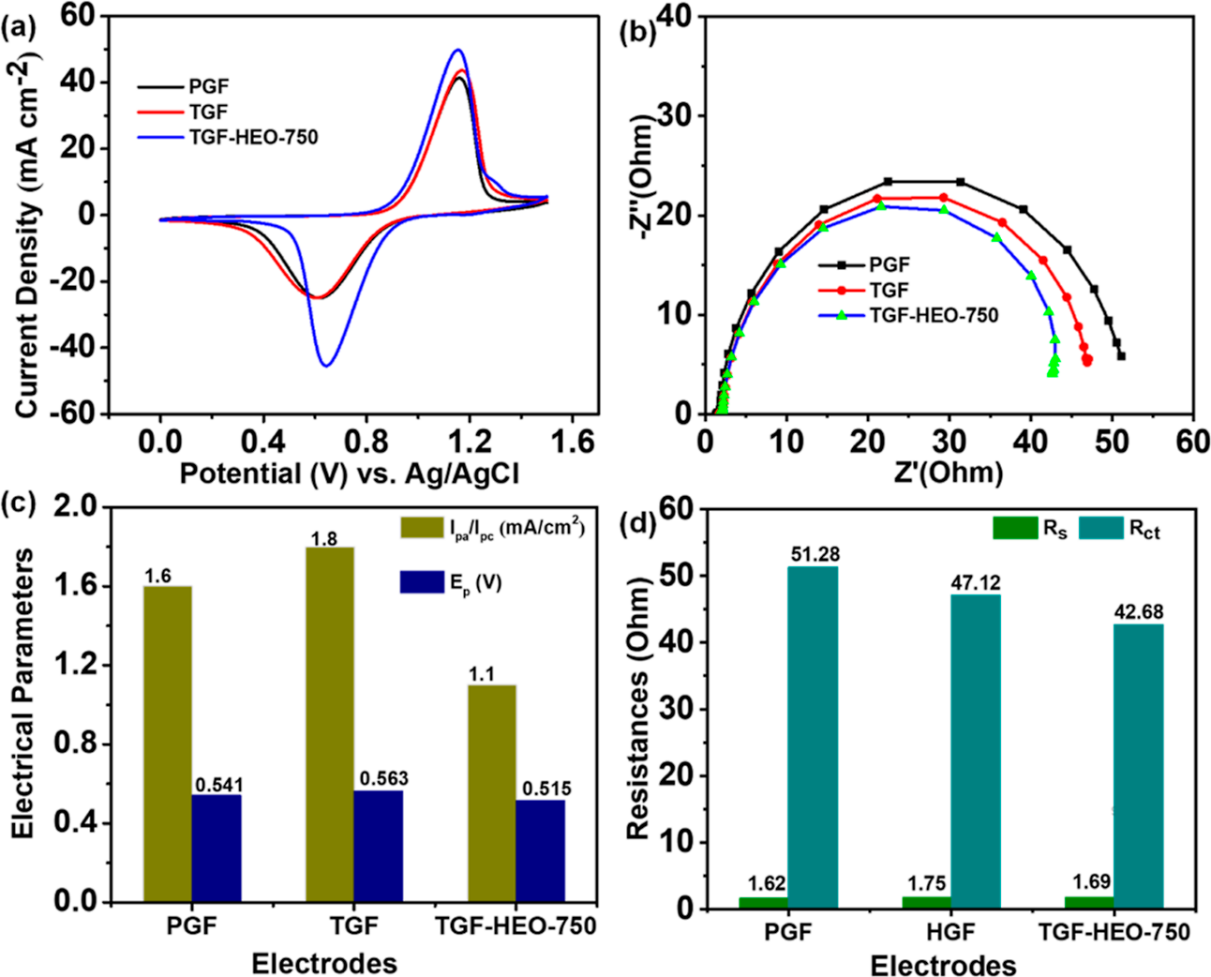
(a,c) CV curves of PGF, TGF, and TGF-HEO-750 and the corresponding values of *I*_pa_/*I*_pc_ and Δ*E*_p_; (b,d) Nyquist plots of the PGF, TGF, and TGF-HEO-750, and the corresponding *R*_s_ and *R*_ct_ values.

Figure 6 shows that the CV and EIS tests were conducted on three different electrodes for PGF, TGF, and TGF with HEO calcined at 750 °C (TGF-HEO-750) to evaluate their electrochemical performance regarding the VO^2+^/VO_2_^+^ redox reaction. In Figure 6a, the CV plots at a 5 mV s^–1^ in a 0.05 M VOSO_4_ + 2 M H_2_SO_4_ electrolyte display two distinct redox peaks for all electrodes, signifying the VO^2+^/VO_2_^+^ couple reaction. Notably, TGF-HEO-750 exhibits the narrowest peak separation (Δ*E*_p_) and the highest peak current density (*J*_pa_ and *J*_pc_), showing superior electrochemical activity. This enhancement is attributed to its abundant active sites, like oxygen vacancies, and a relatively high specific surface area (Table S1). Figure 6b depicts the Nyquist plots of PGF, TGF, and TGF-HEO-750 in a 0.05 M VOSO_4_ + 2 M H_2_SO_4_ solution. These plots span from 100 kHz to 10 mHz at 5 mV with the potential held at open-circuit potential (OCP).^38^ The smaller semicircle radius for TGF-HEO-750 indicates a lower *R*_ct_, which aligns with the CV results in Figure 6a. This alignment underscores the TGF-HEO-750 electrode’s superior electrochemical behavior. In Figure 6c,d, the data extracted from CV and EIS measurements corroborate the findings, emphasizing the TGF-HEO-750 electrode’s enhanced catalytic activity owing to its active sites and reduced charge transfer resistance. The static contact angle between the electrode surface and the aqueous electrolyte can be used to determine an electrode’s wettability, as shown in Figure S11. However, TGF-HEO-750 electrodes (Figure S11c) have a much higher hydrophilicity than that of the listed electrodes.

**Figure 7:**
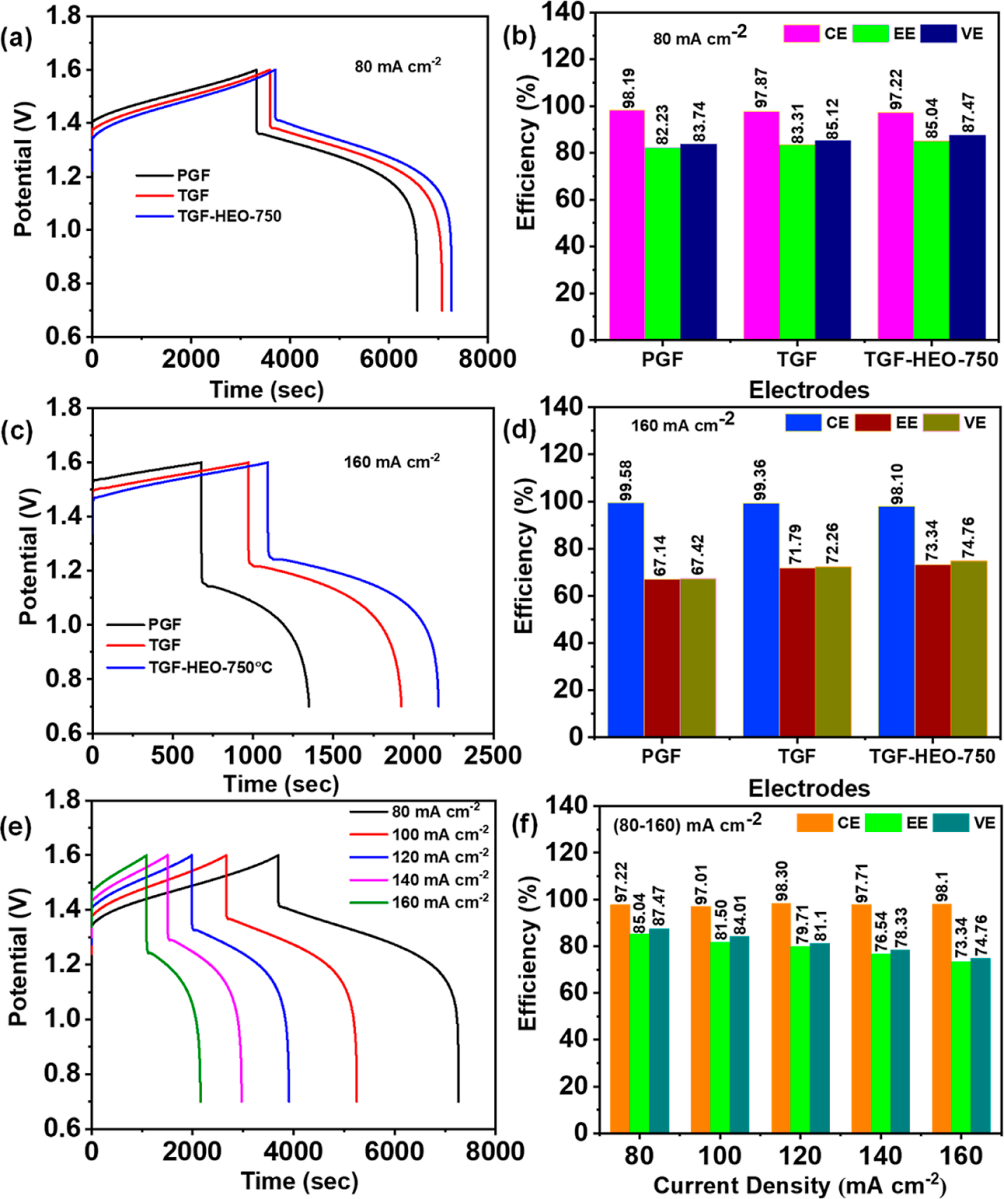
(a,c) Charge–discharge curves for PGF, TGF, and TGF-HEO-750, (b,d) together with the corresponding efficiencies at 80 and 160 mA cm^–2^, respectively (e) TGF-HEO-750 at various current densities, and (f) estimated efficiency values.

**Figure 8:**
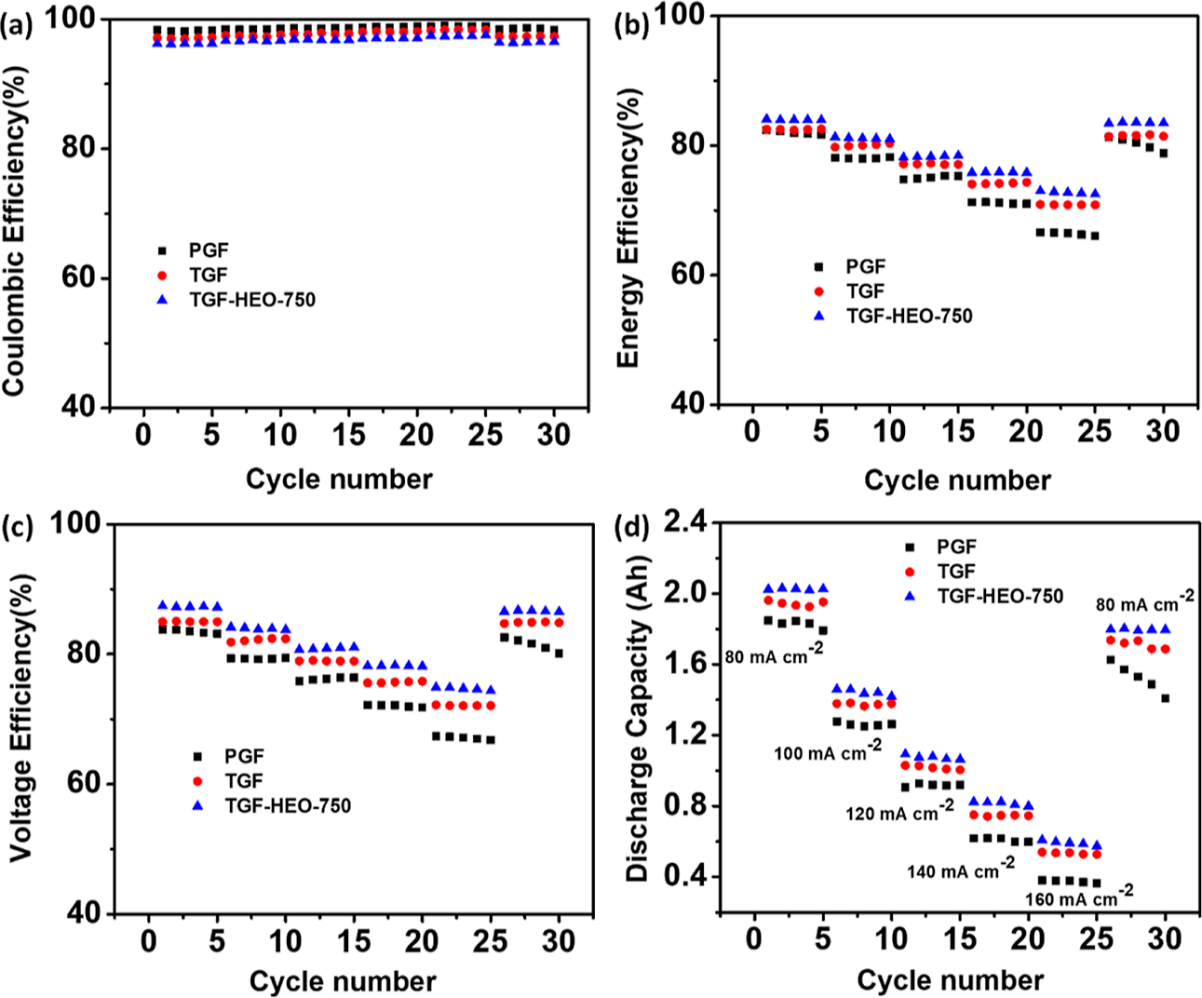
(a) CE, (b) EE, (c) VE, and (d) discharge capacity of the PGF, TGF, and TGF- HEO-750 electrodes with cycle numbers at different current densities of 80–160 mA cm^–2^.

Figure 7a,c compares the charge–discharge of the cells with PGF, TGF, and TGF-HEO-750 electrodes, recorded at the same current density of 80 and 160 mA cm^–2^. The results show that the cell with the TGF-HEO-750 electrode has a significantly lower reaction overpotential in the related charge–discharge processes, a longer discharge duration, a smaller charge voltage, and a greater discharge voltage than that of the cells with PGF and TGF, and thus, it achieves the best performance. The efficiencies obtained from Figure 7a,c are shown in Figure 7b,d, respectively. Notably, the TGF-HEO-750 electrode obtains the best efficiencies of all the investigated samples, with 97.22% CE, 87.47% VE, and 85.04% EE at a current density of 80 mA cm^–2^, respectively. The HGF-HEO-750 electrode exhibits a commendable electrochemical performance, even when subjected to a high current density of 160 mA cm^–2^. As indicated in Figure 7c, the electrodes’ CE, VE, and EE are 98.10, 74.76, and 73.34%, respectively. Consequently, the annealing treatment induces the creation of new fluorite HEO and numerous oxygen vacancies, essential for fostering synergistic effects that enhance the electrochemical performance. Figure 7e depicts the charge–discharge patterns of the cell with the HGF-HEO-750 electrode at various current densities ranging from 80 to 160 mA cm^–2^; Figure 7f summarizes the resulting efficiencies. With increased current density, the CE rises because of inhibited crossover. On the contrary, the VE and EE fall as polarization drives the charging voltage toward the voltage cutoff, which is designed to avoid water splitting.^38,60^ Moreover, Figure S16 shows the charge–discharge of PGF, TGF, TGF-HEO-750, and both sides of TGF-HEO-750 at 80 mA cm^–2^. Figure 8 shows the charging–discharging rate capacity, discharge capacity, and capacity recovery of the electrodes. As seen in Figure 8a, the CE values for the individual electrodes are almost similar at the same current density. The matching values rise slightly with rising current density because the crossover of vanadium ions through Nafion membranes takes less time.^61^ However, a fast charge–discharge results in a considerable increase in the overpotential and a significant drop in the EE and VE, as shown in Figure 8b,c, respectively. The dissolution tests using UV–vis spectroscopy reveal the distinct color changes in vanadium electrolytes. Figure S17a illustrates the UV spectra obtained from vanadium electrolytes in our laboratory. The various oxidation states and absorbance peaks of V(II) (violet, 855 nm), V(III) (green, 610 nm), V(IV) (blue, 765 nm), and V(V) (yellow, 389.09 nm) are depicted in Figure S17c. The unique color variations enable UV spectroscopy to serve as a distinctive fingerprint for vanadium ions.^62,63^

The values of EE and VE of the cell constructed utilizing the TGF-HEO-750 electrode are superior to those of PGF and TGF at the same current density. In addition, the discharge capacities of the TGF-HEO-750 electrode are much larger than those of PGF and TGF cells at all applied currents, as shown in Figure 8d. The difference is significant even at a high current density of 160 mA cm^–2^. When the current density is reduced from 160 to 80 mA cm^–2^, the TGF-HEO-750 electrode performs almost identically in terms of EE, VE, and discharge capacity, demonstrating that the electrochemical resistance of the TGF-HEO-750 electrode in the electrolyte is favorable.

**Figure 9:**
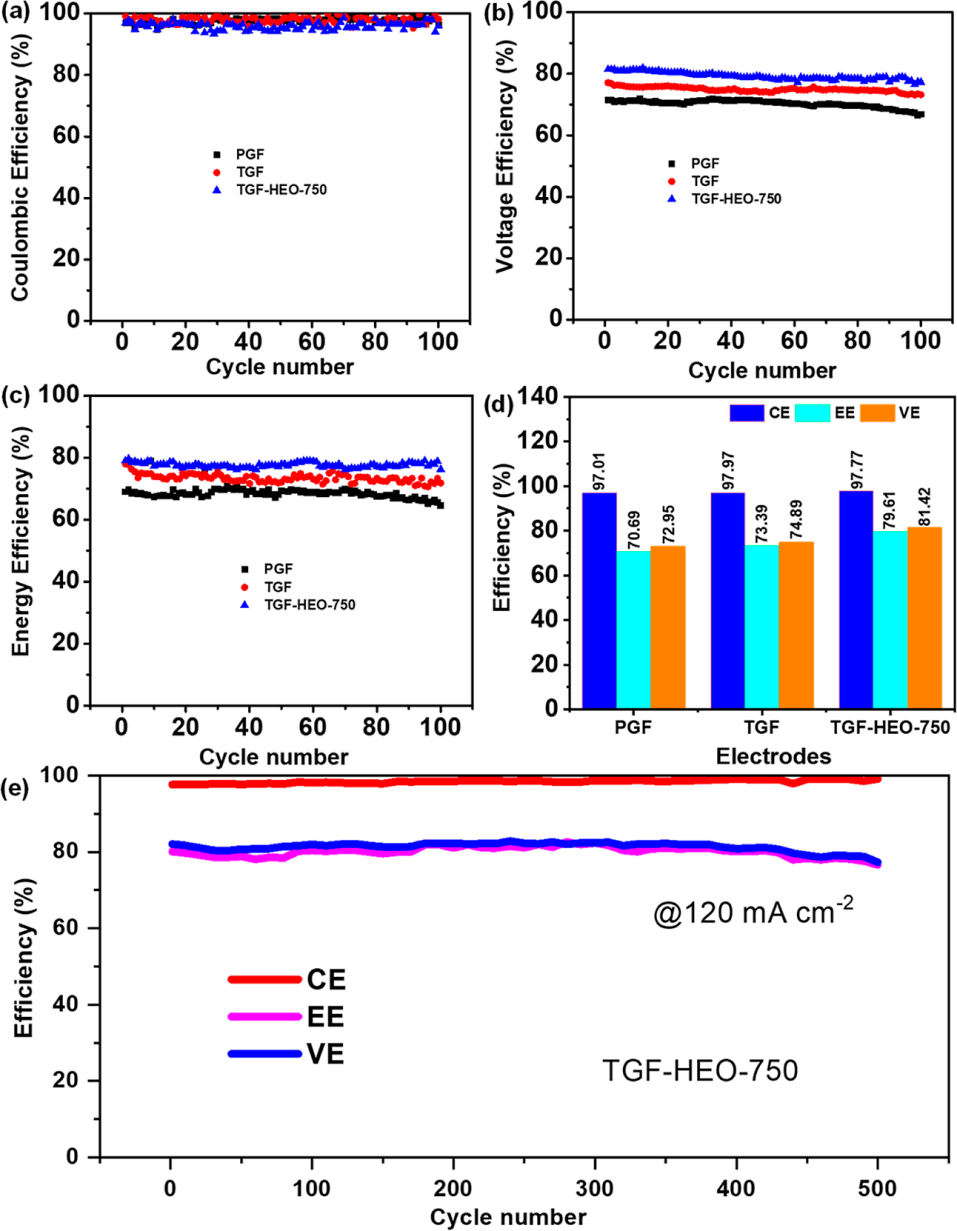
Cycle performances of VRFB single cells with PGF, TGF, and TGF-HEO-750 electrodes: (a) CE, (b) VE, (c) EE, and (d) efficiency values estimated from 100 cycles at 120 mA cm^–2^ and (e) stability of TGF-HEO-750 for 500 cycles at 120 mA cm^–2^.

Long-term cycling experiments were carried out to assess the operation stability of the PGF, TGF, and TGF-HEO-750 electrodes after 100 cycles at 120 mA cm^–2^, as illustrated in Figure 9. The CE values of the PGF, TGF, and TGF-HEO-750 electrodes are approximately identical, as shown in Figure 9a. This suggests that the impact of self-discharge and side reactions (such as hydrogen evolution and oxygen evolution)^64^ on the GFs or TGF-HEO-750 electrodes is similar. On the other hand, the VE and EE of all samples under the long-term cycling tests are shown in Figure 9b,c, respectively, indicating that TGF-HEO-750 is almost consistent over 100 cycles. Figure 9d summarizes the efficiency values estimated from 100 cycles at 120 mA cm^–2^. The TGF-HEO-750 electrode after 100 cycles ensures the electrode’s good chemical stability and electrochemical robustness. Moreover, it also shows outstanding stability for 500 cycles at 120 mA cm^–2^, as shown in (Figure 9e).

The SEM/EDS images of the electrode before and after the charge–discharge cycles are shown in Figure S12. The XPS shows the elemental composition and chemical bond information after multiple charge–discharge cycles, as shown in Figure S13. It demonstrates the presence of all six cation species on the surface of the GF, including the O 1s and C 1s spectra. Due to the harsh acidic environment, some elements change in oxidation state, and their satellite peaks disappear. Generally, XPS and SEM/EDS images indicate the cation species present on the GF electrode after several charge–discharge cycles. Furthermore, Figure S14a,b shows the CV and EIS curves after the charge–discharge test was conducted for the TGF and TGF-HEO-750 samples. Figure S14c shows the fitting results obtained from Figure S14a,b. Lastly, the XRD patterns before and after charge–discharge were performed to verify the crystalline nature of the cathode material TGF-HEO-750, as shown in Figure S15. Well-defined diffraction peaks were observed before the charge–discharge test (Figure S15a). Following the charge–discharge test, three weak diffraction peaks in the XRD pattern were observed at approximately 34, 47, and 57°. Additionally, the carbon peaks were more prominent than the observed diffraction peaks.

The remarkable performance can be due to the following factors: (1) the formation of a new fluorite single-phase structure during annealing, (2) plenty of oxygen vacancies can provide electroactive sites and enough pathways for electron transfer, increase the electron transport speed within the material, and enhance the electrochemical performance of VRFBs. (3) The metal cations are uniformly distributed across the surface of the GF. (4) High current signals indicate substantial electron transfer due to the efficient use of electroactive La, Bi, Ce, Zr, Mo, and W species, resulting in improved charge storage capacity. (5) HEO contains a high concentration of trivalent, tetravalent, and hexavalent ions. This may enhance the number of electrons in the vanadium storage process and their capacity. (6) The distinctive structure of HEO can resist high volumetric changes after repeated charge–discharge processes. (7) Stabilization of entropy modifies HEOs’ phase stability and functional properties. (8) Hydrophilicity provided active sites and improved electrolyte accessibility.

**Scheme 3:**
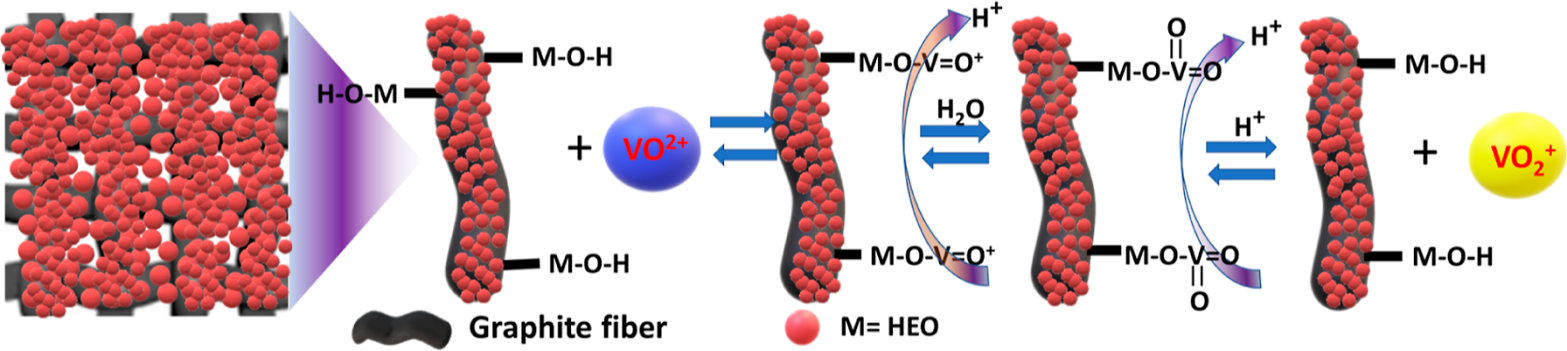
Proposed Catalytic Reaction Mechanisms for the Redox Reaction Involving VO_2_^+^/VO^2+^ Using HEO-750 Modification on the TGF Surface

Finally, the catalytic effect of the HEO surface on the VO_2_^+^/VO^2+^ redox reaction is proposed in Scheme 3. Skyllas-Kazacos et al.^65,66^ suggested a potential mechanism involving key steps during the charging process: (1) diffusion of VO^2+^ ions from the electrolyte to the surface of the electrode; (2) adsorption of VO^2+^ and ion exchange on the functionalized HEO surface; (3) electron-transfer reaction from VO^2+^ to form VO_2_^+^, followed by diffusion of vanadium ions back into the solution.^66^ This process is reversed during discharge.

The VRFB cell, employing the TGF-HEO-750 electrode, demonstrates an EE of 73.34% at 160 mA cm^–2^, surpassing the performance of the VRFBs with the GF electrodes modified by alternative electrocatalysts. In comparison to various related studies summarized in Table S5, the electrocatalytic performance of the synthesized TGF-HEO-750 as the positive electrode for VRFB significantly outperforms those of previously reported catalysts.

## 4 Conclusions

A simple surfactant-assisted hydrothermal technique was employed to synthesize single-phase fluorite HEO nanoparticle electrocatalysts as an VRFB’s anode. We investigated the effect of calcination temperatures, hydrothermal treatment, and the inclusion of several metal cations into single-phase crystal formation of HEO (BiZrMoWCeLa)O_2_ nanoparticles used as an electrocatalyst for VRFB, which can facilitate the vanadium redox pairs’ redox processes. The entropy-driven single-phase stabilization brings considerable advantages for retaining the storage capacity and greatly enhances the cycling stability of the VRFBs. Moreover, observations suggest that the electrochemical performance of the HEO depends on the specific metal cations present and can be improved by adjusting the elemental composition. As a result, the HEO-750 sample displays superior reversibility and electrocatalytic activity toward the VO^2+^/VO_2_^+^ and the V^3+^/V^2+^ redox couples. Consequently, the charge–discharge tests demonstrate that a VRFB employing the TGF-HEO-750 electrode shows excellent CE, VE, and EE of 97.22, 87.47, and 85.04% at 80 mA cm^–2^ and 98.10, 74.76, and 73.34% at a higher current density of 160 mA cm^–2^, respectively. Furthermore, the durability of the TGF-HEO-750 electrode exhibits an outstanding operational lifespan of 500 cycles. The energy and voltage efficiencies outperformed those of VRFB cells assembled with the PGF and TGF. This comprehensive research proves to be advantageous for the advancement of high-performance electrodes in VRFBs. It illuminates the path for future exploration of HEOs in the context of VRFB applications.

## Supporting Information Available

SEM images, BET measurement and results, EDS, FTIR spectrum, XRD patterns, XPS O 1s spectra with fitting results, CV curves with fittings, CV curve different scan rates, LSV with fittings, in situ Raman, EIS with fittings, contact angle measurement, SEM/EDS before and after charge–discharge, XPS spectra after charge–discharge cycles, CV and EIS with fitting after charge–discharge cycles, XRD patterns after charge–discharge cycles, charge–discharge profiles, UV–vis spectra, and vanadium electrolyte color, and comparison of electrode performance of TGF-HEO-750 with those of previously reported materials (PDF)
