# Supplementary material for: High-Entropy Oxide of (BiZrMoWCeLa)O2 as a Novel Catalyst for Vanadium Redox Flow Batteries
Source: ACS Appl Mater Interfaces. 2024 Feb 20;16(8):10019–32. doi: 10.1021/acsami.3c15783 (PMC10910445; doi:10.1021/acsami.3c15783)
Supplement: Supplementary file 1 — am3c15783_si_001.pdf [file am3c15783_si_001.pdf]

Supporting Information

High-entropy Oxide of $(BiZrMoWCeLa)O_2$ as Novel Catalyst for Vanadium Redox Flow Batteries

Aknachew Mebreku Demeku,[1] Daniel Manaye Kabtamu,[#1,2] Guan-Cheng Chen,[1] Yun-Ting Ou,[1] Zih-Jhong Huang,[1] Ning-Yih Hsu,[3] Hung-Hsien Ku,[3] Yao-Ming Wang,[4] Chen-Hao Wang*[1,5,6]

[1]*Department of Materials Science and Engineering, National Taiwan University of Science and Technology, Taipei 106335, Taiwan*

[2]*Department of Chemistry, Debre Berhan University, Po.Box: 445, Debre Berhan, Ethiopia*

[3] *Chemistry Division, National Atomic Research Institute, 325207, Taoyuan, Taiwan*

[4] *Maritime Innovation & Industry Promotion Department, Metal Industries Research & Development Centre, Kaohsiung 811160, Taiwan*

[5] *Hierarchical Green-Energy Materials (Hi-GEM) Research Center, National Cheng Kung University, Tainan 701401, Taiwan*

[6] *Center of Automation and Control, National Taiwan University of Science and Technology, Taipei 106335, Taiwan*

**Corresponding author:* chwang@mail.ntust.edu.tw *(Prof. Chen-Hao Wang)* Fax: +886-2-2737-6544; Tel: +886-2-2730-3715

[#]*Co-corresponding author:* danielmanaye@gmail.com *(Dr. Daniel M. Kabtamu)*

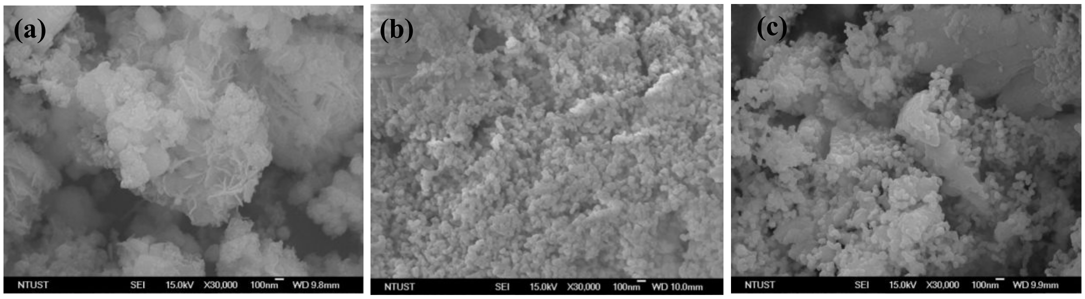

Figure S1 SEM images of the (a) HEO-550, (b) HEO-750, and (c) HEO-900.

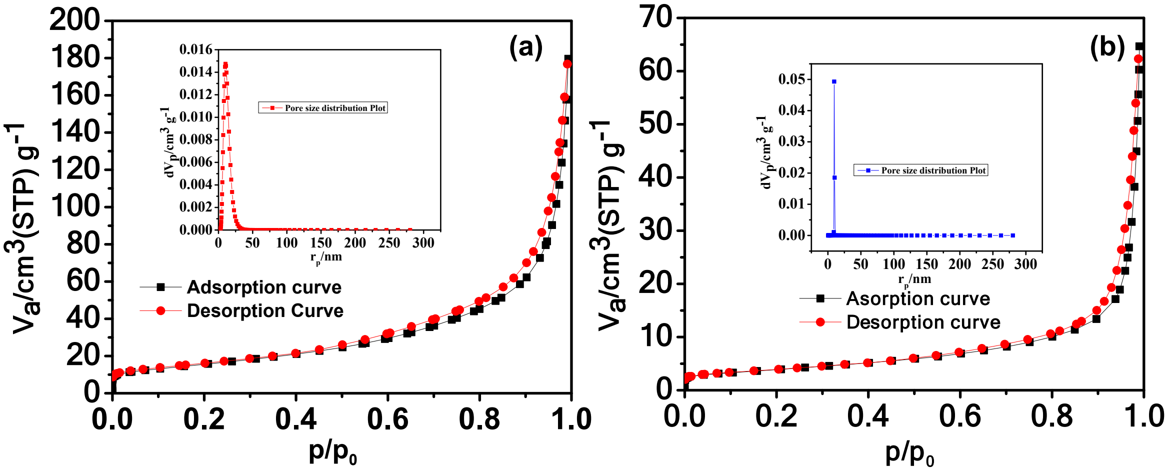

Figure S2 (a) Nitrogen adsorption-desorption isotherms of the BET measurement as-synthesized HEO-750, (b) HEO-900.

Table S1 BET and BJH measurement results obtained from **Figure S2 (a-b).**

Electrocatalysts	Surface area (m^2 g^{-1})	Porosity (cm^3 g^{-1}) Total pore volume	Mean pore diameter (nm)
**HEO-750**	54.743	0.251	18.34
**HEO-900**	13.125	0.098	29.81

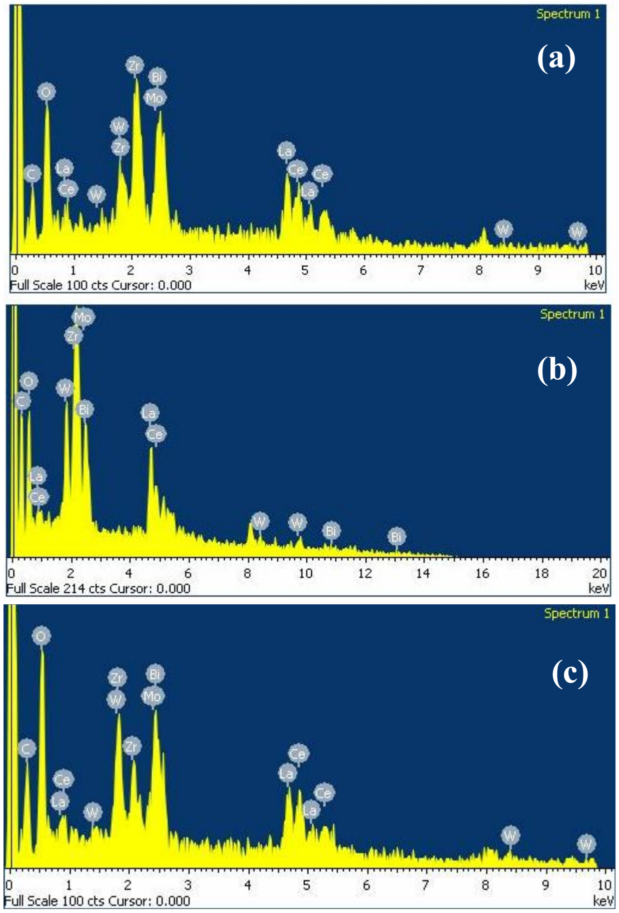

(a)

Element	Weight%	Atomic%
C K	9.06	33.92
O K	14.59	41.04
Zr L	14.87	7.33
Mo L	1.30	0.61
La L	18.82	6.10
Ce L	18.42	5.92
W M	4.66	1.14
Bi M	18.27	3.93
Totals	100.00	

(b)

Element	Weight%	Atomic%
C K	11.45	36.67
O K	18.69	44.96
Zr L	6.76	2.85
Mo L	2.94	1.18
La L	15.33	4.25
Ce L	17.27	4.74
W M	10.69	2.24
Bi M	16.88	3.11
Totals	100.00	

(c)

Element	Weight%	Atomic%
C K	18.62	48.84
O K	18.45	36.35
Zr L	14.91	5.15
Mo L	3.40	1.12
La L	11.57	2.63
Ce L	9.90	2.23
W M	9.53	1.63
Bi M	13.61	2.05
Totals	100.00	

Figure S3 EDS graph of the (a) HEO-550, (b) HEO-750, and (c) HEO-900.

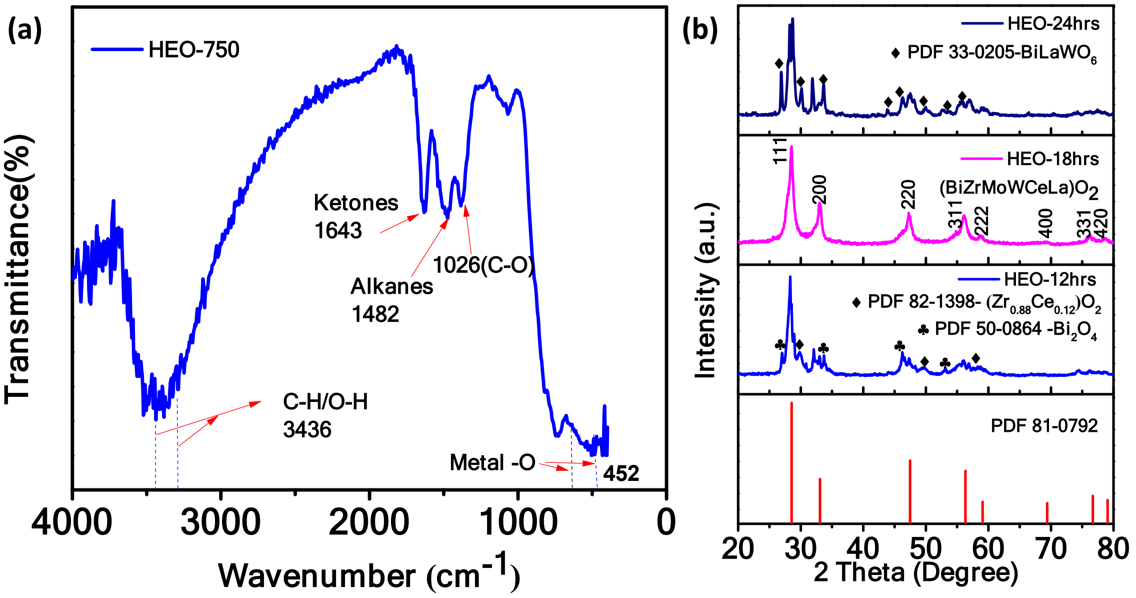

Figure S4 (a) The FTIR spectrum and (b) XRD patterns of different hydrothermal treatments of (12, 18, and 24) hrs of HEO-750 nanoparticles.

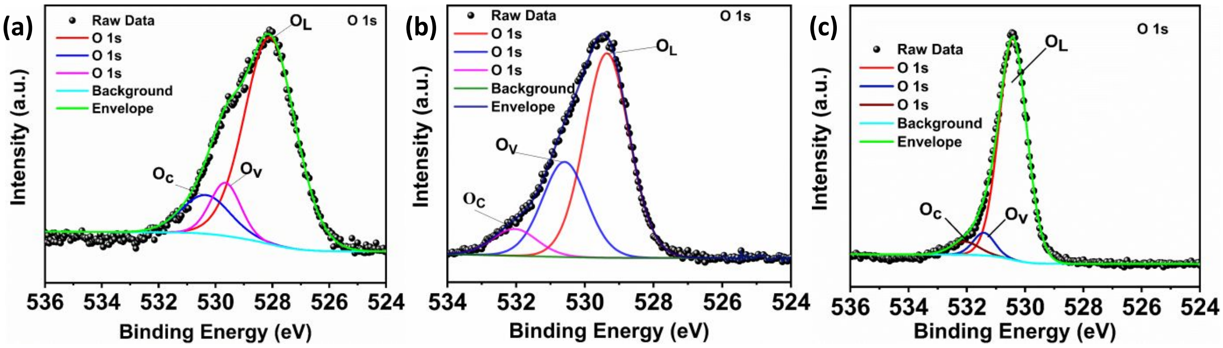

Figure S5 The O 1s XPS spectra of (a) HEO-550, (b) HEO-750, and (c) HEO-900.

Table S2 The fitting results of XPS O 1s spectra obtained from **Figure S5**.

O1s	Lattice oxygen (O_L)	Oxygen vacancy (O_v)	Chemisorbed oxygen (O_C)
HEO-550	29.23 %	13.42 %	57.34 %
HEO-750	3.69 %	17.74 %	78..56 %
HEO-900	84.9 %	6.91 %	5.3 %

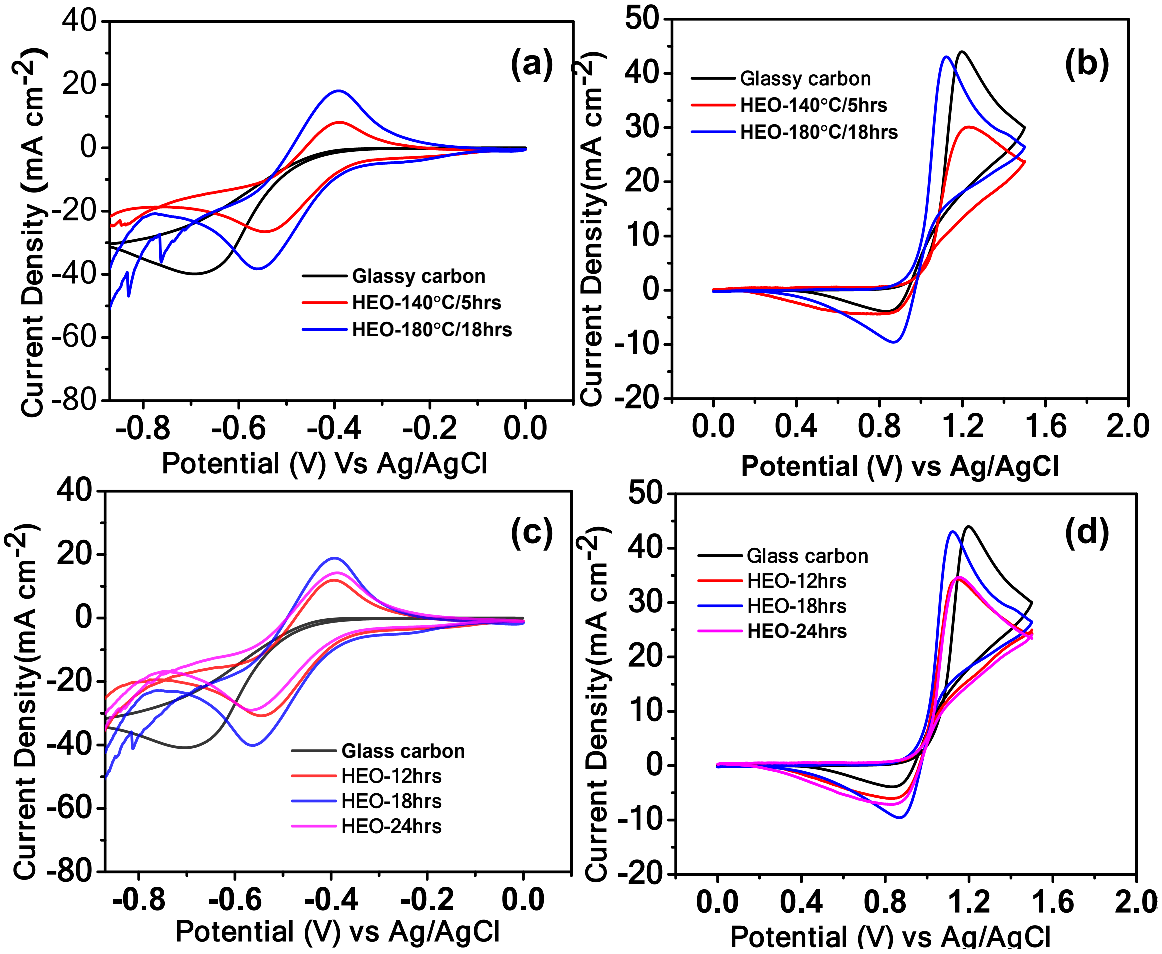

Figure S6 The cycle voltammetry (CV) curves of (a, b) HEO-(140°C, 180°C)/(5, 18) hrs, (c, d) HEO-(12, 18, 24) hrs in Anolyte and Catholyte, respectively in the electrolyte solutions of 1.6 M $VOSO_4$ + 4.6 M H_2SO_4 at the scan rate of 10 mV s^{-1}.

Table S3 The cyclic voltammetry results obtained from **Figure S6.**

Types		Electrodes				
		Glassy carbon	HEO-140°C /5hrs	HEO-180°C /12hrs	HEO-180°C /18hrs	HEO-180°C /24hrs
Negative side	I_{pa}/I_{pc}(mA/cm^2)	-	0.88	1.13	1.02	1.23
	ΔE_p (mV)	-256	-148	-170	-169	-180
Positive side	I_{pa}/I_{pc}(mA/cm^2)	1.7	1.92	1.62	1.43	1.59
	ΔE_p (mV)	286	278	251	233	245

The CV curves of the HEO-750 electrode for the VO^{2+}/VO_2^+ were conducted at different scan rates, as shown in **Figure S7a**. The peak current (i_{pa} and i_{pc}) as a function of the square root of the scanning rate for the HEO-750 electrode (**Figure S7b**). The cathodic and anodic peak current densities of the VO^{2+}/VO_2^+ redox pair are proportional to the square root of the scan rate, indicating that the electrochemical behavior of the redox couple at the electrode was diffusion controlled.[1] Because peak current versus square root of scan rate is nonlinear, CV is not the best way to determine kinetic parameters. Therefore, the kinetic parameters were determined using the rotating disc electrode (RDE) method, i.e., LSV curves. The electrode reaction rates of HEO-750 were investigated using linear sweep voltammetry (LSV), as shown in **Figure S8**. The rotation speed was varied from 200 rpm to 2000 rpm (**Figure S8a**). LSV was scanned from 0 V to 2 V on the positive side versus Ag/AgCl at the scan rate of 2 mV s^{-1}. The linear relationship between limiting current, i_L, and the square root of rotation speed ($\omega^{1/2}$) is shown in **Figure S8b**, which reveals the good electrochemical activity of HEO-750. **Figure S8b** plots the Levich behavior of the HEO-750 assembled electrodes governed by the equation below.[2, 3]

$$i_L = 0.62nFAD^{2/3}\omega^{1/2}\upsilon^{-1/6}C_0 \quad (1)$$

$$i_k = nFAk^0C_o = nFAk^0C_o\exp\left(\frac{-\alpha nF(E-E^{0'})}{RT}\right) \quad (2)$$

From the slopes of the curves given in **Figure S8b**, the calculated diffusion coefficients for $VOSO_4$ using **Equation 1** are 2.1246×10^{-5} cm^2 s^{-1}. **Figure S8c** is obtained by taking the logarithm of the reciprocal of the y-intercepts of **Figure S8d**. The values of k^0 were obtained from i_0 using

Equation 2. The exchange current and estimated standard rates constant are 7.75×10^{-3} A and 2.5×10^{-4} cm s^{-1}, respectively.

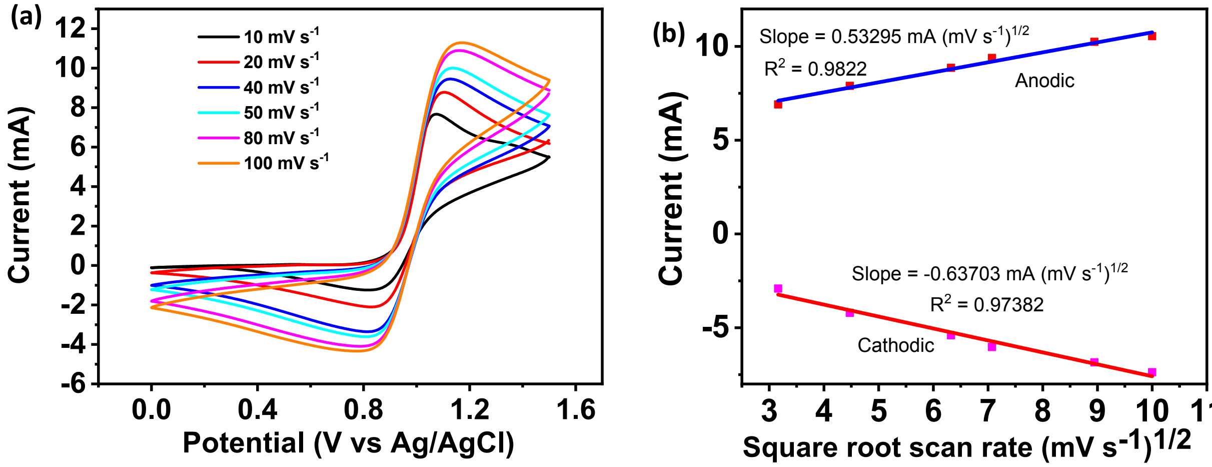

Figure S7 (a) CV curves of HEO-750 electrode in 1.6 M $VOSO_4$ in 4.6 M H_2SO_4 electrolyte at scan rates ranging from 10 to 100 mV s^{-1}. (b) Peak current (i_{pa} and i_{pc}) as a function of the square root of the scanning rate for the HEO-750 electrode.

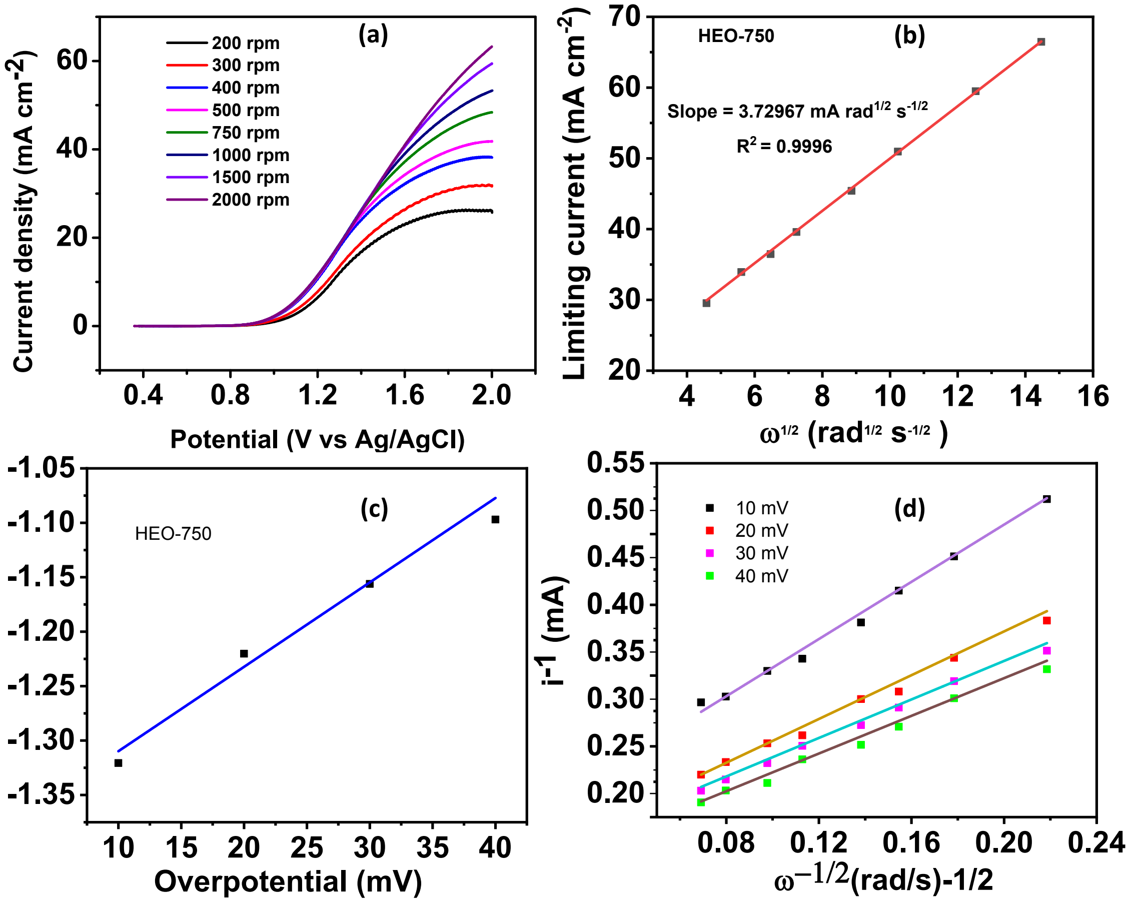

Figure S8 (a) Anodic polarization curves of HEO-750 electrode at different electrode rotating speeds, (b) Linear fitted Levich plots of the limiting current density (i_L) versus the square root of rotation rates ($\omega^{1/2}$), (c) Linearly fitted plots of log i_k as a function of the overpotential (η) (d). Linearly fitted Koutecky-Levich plots of i^{-1} versus $\omega^{-1/2}$.

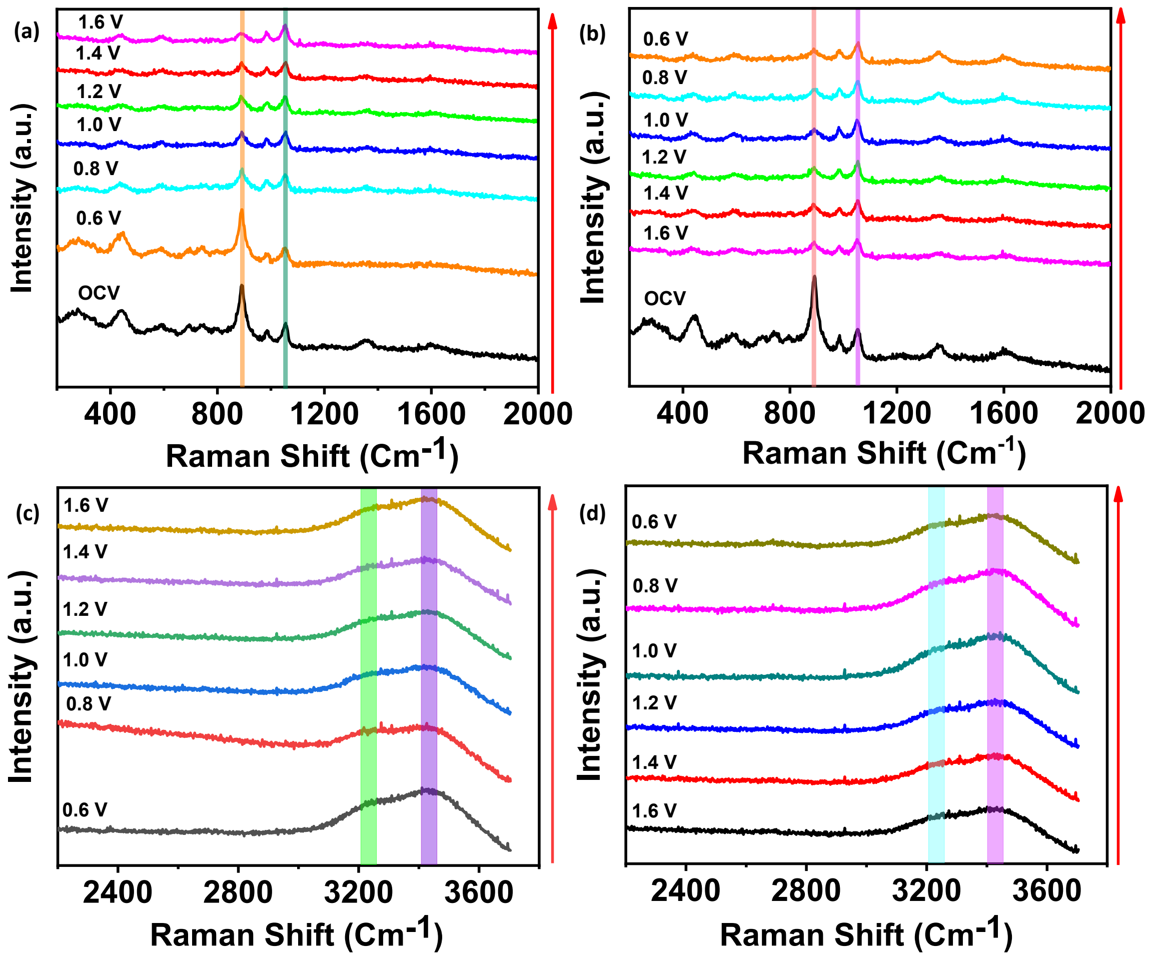

Figure S9 Potential-dependent in situ Raman spectra measured at (a, b) low wavenumber and (c, d) high wavenumber regimes of HEO-750 in 0.05 M $VOSO_4$ + 2 M H_2SO_4.

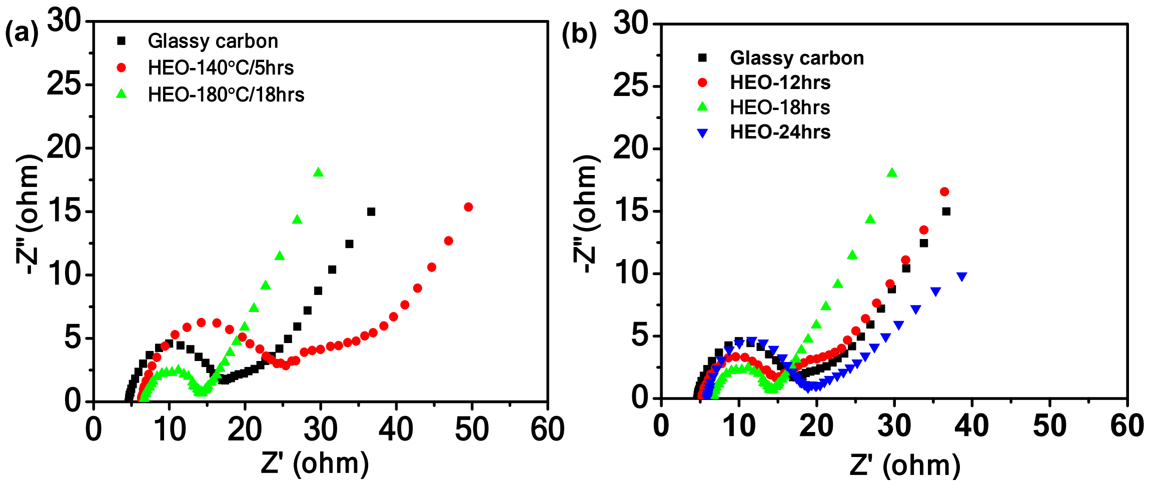

Figure S10 Nyquist plots of of (a) HEO-(140°C, 180°C)/(5, 18) hrs and(b) HEO-(12, 18, 24) hrs in the electrolyte solutions of 1.6 M $VOSO_4$ + 4.6 M H_2SO_4 at a polarization potential of 1.0 V.

Table S4 EIS results obtained from **Figures S10a** and **S10b.**

**Resistances**	**Electrodes**				
	Glassy carbon	HEO-140°C/5hrs	HEO-180°C/12hrs	HEO-180°C/18hrs	HEO-180°C/24hrs
**Rs (Ω)**	4.59	6.23	5.65	6.53	5.92
**Rct (Ω)**	11.39	13.93	10.55	7.66	10.87

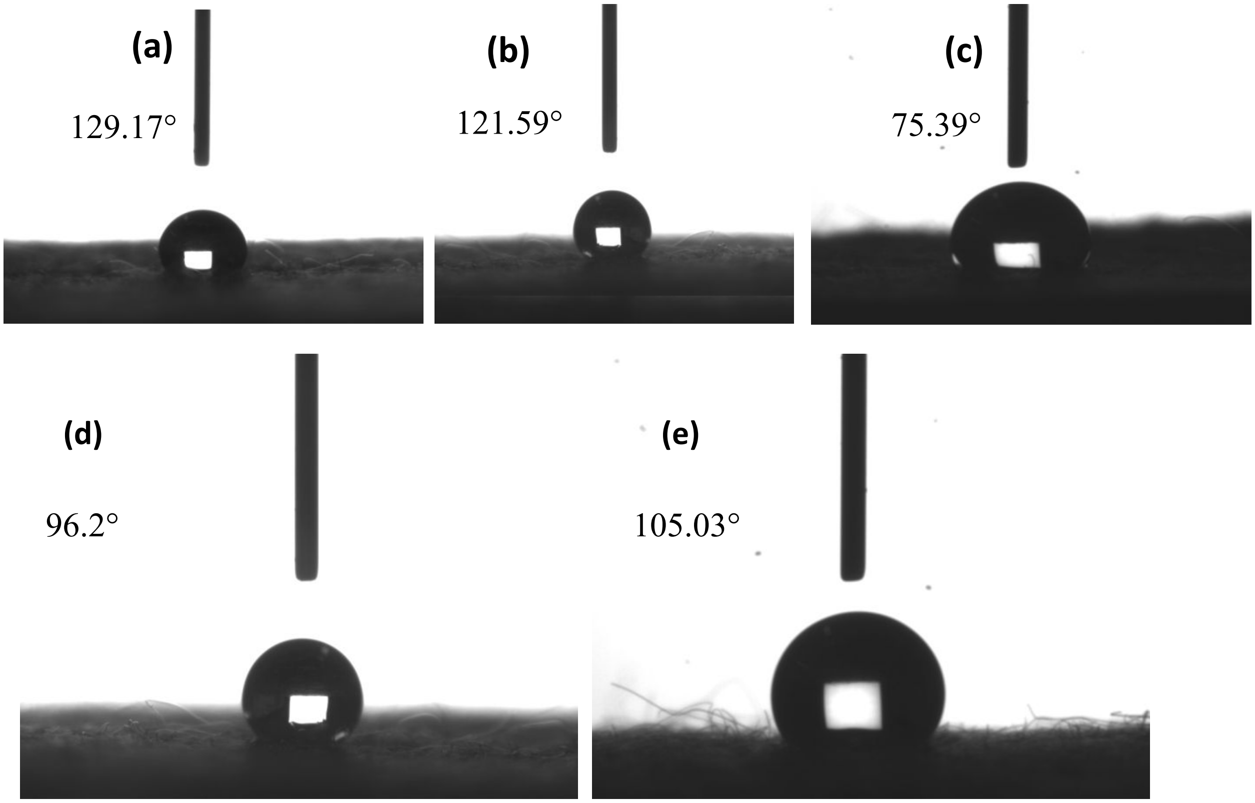

Figure S11 Photographs of the contact angle measurements on (a) PGF, (b) TGF, (c) TGF-HEO-750, (d) TGF-HEO-550, and (e) TGF-HEO-900.

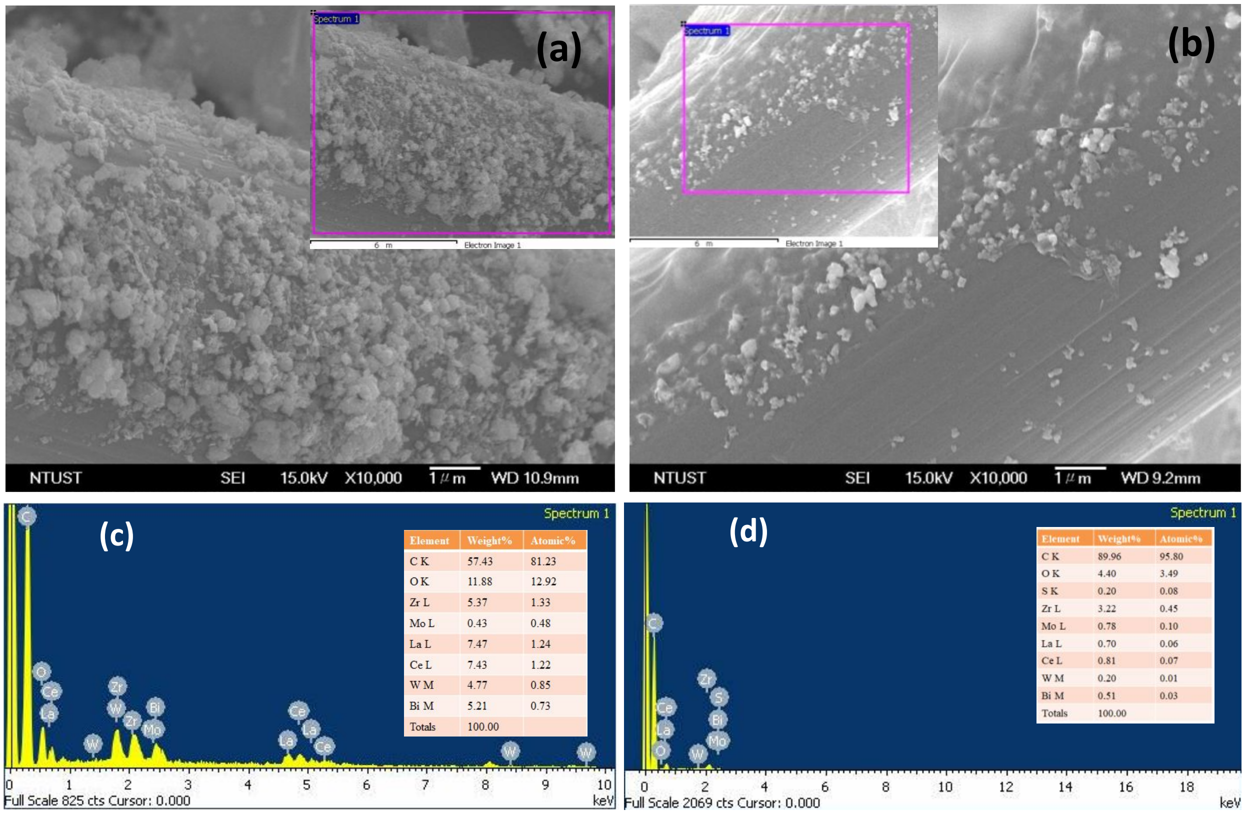

Figure S12 SEM images of TGF-HEO-750 (a) before charge-discharge, (b) after charge-discharge, and (c, d) the elemental mapping of TGF-HEO-750 before and after charge-discharge, respectively.

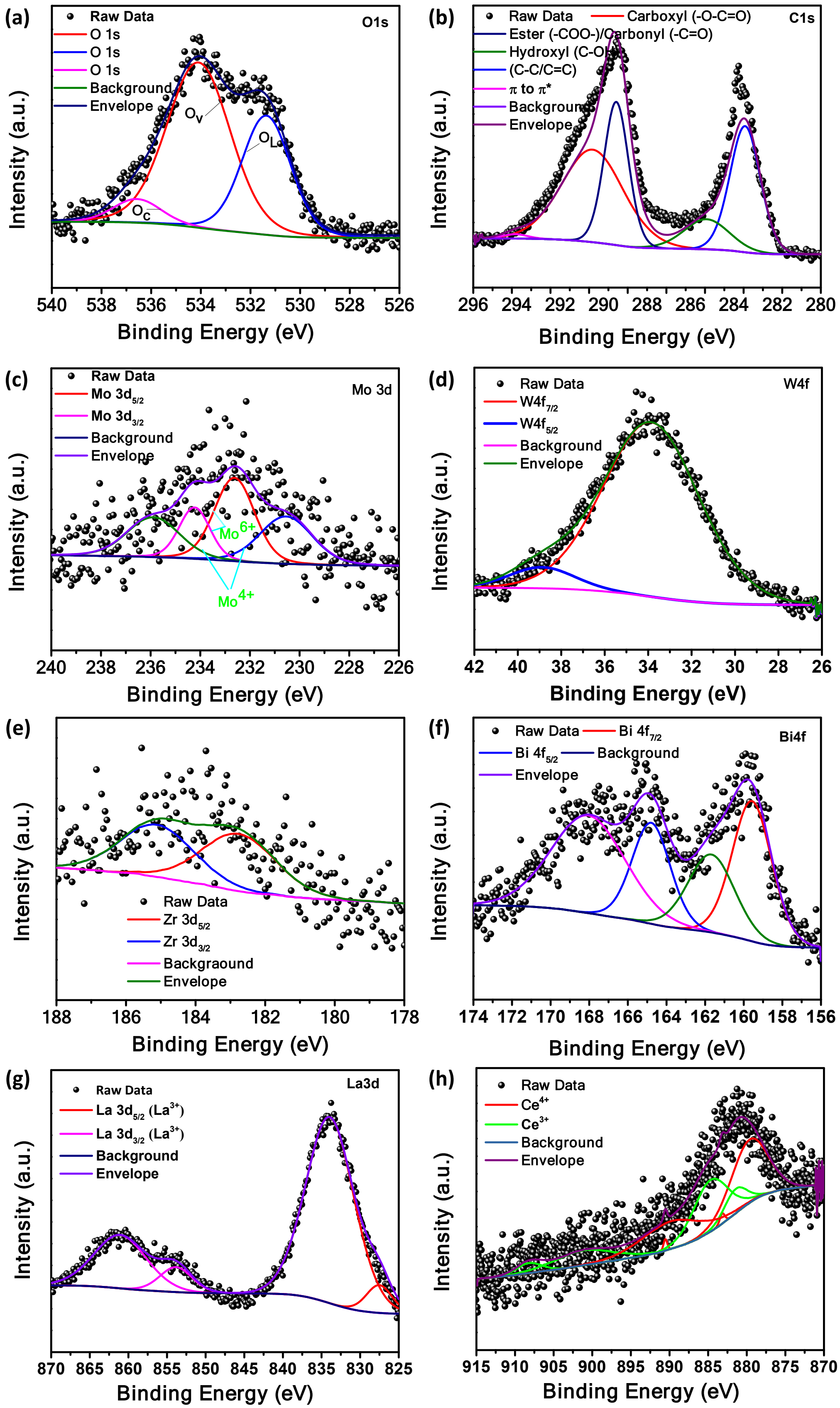
(a)
Raw Data
O 1s
O 1s
O 1s
Background
Envelope
O1s
O_V
O_L
O_C
Intensity (a.u.)
Binding Energy (eV)
(b)
Raw Data
Carboxyl (-O-C=O)
Ester (-COO-)/Carbonyl (-C=O)
Hydroxyl (C-OH)
(C-C/C=C)
π to π*
Background
Envelope
C1s
Intensity (a.u.)
Binding Energy (eV)
(c)
Raw Data
Mo $3d_{5/2}$
Mo $3d_{3/2}$
Background
Envelope
Mo 3d
Mo^{6+}
Mo^{4+}
Intensity (a.u.)
Binding Energy (eV)
(d)
Raw Data
$W4f_{7/2}$
$W4f_{5/2}$
Background
Envelope
W4f
Intensity (a.u.)
Binding Energy (eV)
(e)
Raw Data
Zr $3d_{5/2}$
Zr $3d_{3/2}$
Backgraound
Envelope
Intensity (a.u.)
Binding Energy (eV)
(f)
Raw Data
Bi $4f_{7/2}$
Bi $4f_{5/2}$
Background
Envelope
Bi4f
Intensity (a.u.)
Binding Energy (eV)
(g)
Raw Data
La $3d_{5/2}$ (La^{3+})
La $3d_{3/2}$ (La^{3+})
Background
Envelope
La3d
Intensity (a.u.)
Binding Energy (eV)
(h)
Raw Data
Ce^{4+}
Ce^{3+}
Background
Envelope
Intensity (a.u.)
Binding Energy (eV)

Figure S13 XPS spectra (a) O1s, (b) C1s, (c) Mo3d, (d) W4f, (e) Zr3d, (f) Bi4f, (g) La3d, (h) Ce3d of TGF-HEO-750 electrode after charge-discharge cycles.

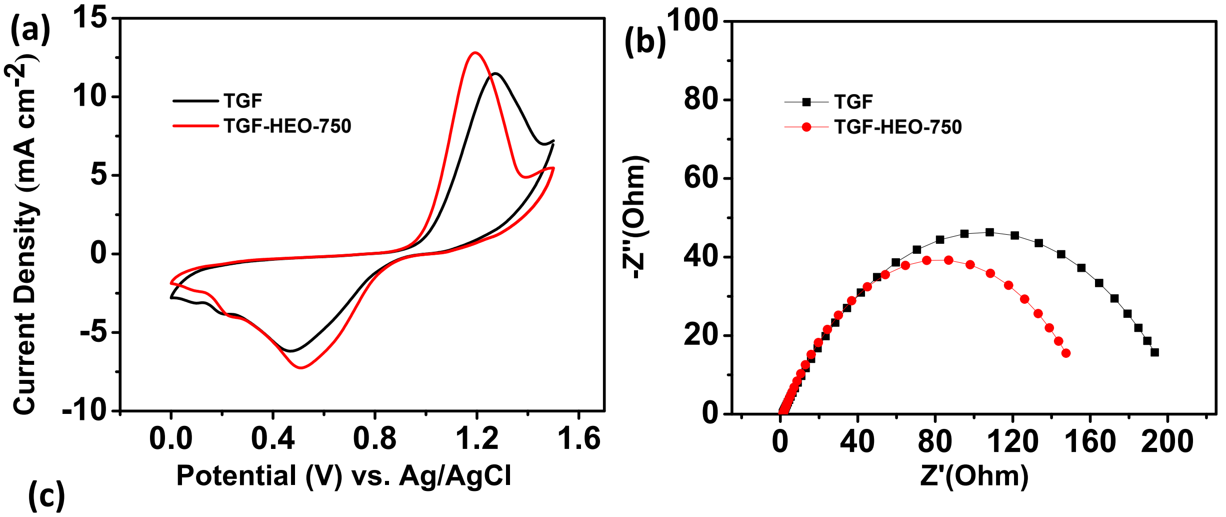

(c)

Electrodes	J_{pa} (mA cm^{-2})	J_{pc} (mA cm^{-2})	$\lvert J_{pa}/J_{pc}\rvert$	E_{pa} (V)	E_{pc} (V)	ΔE_p	R_s (Ω)	R_{ct} (Ω)
TGF	11.4012	-6.012	1.896	1.2711	0.4741	0.797	1.127	209
TGF-HEO-750	12.956	-7.2412	1.789	1.1947	0.5185	0.676	1.305	160

Figure S14. (a, c) the CV curves of TGF and TGF-HEO-750 and the corresponding values of I_{pa}/I_{pc} and ΔE_p with a scan rate of 5 mV s^{-1}; (b, c) the Nyquist plots of the TGF and TGF-HEO-750, and the corresponding R_s and R_{ct} values at open-circuit potential 5 mV, towards VO^{2+} /VO_2^+ in 0.05 M $VOSO_4$ + 2 M H_2SO_4 solutions after charge-discharge cycles.

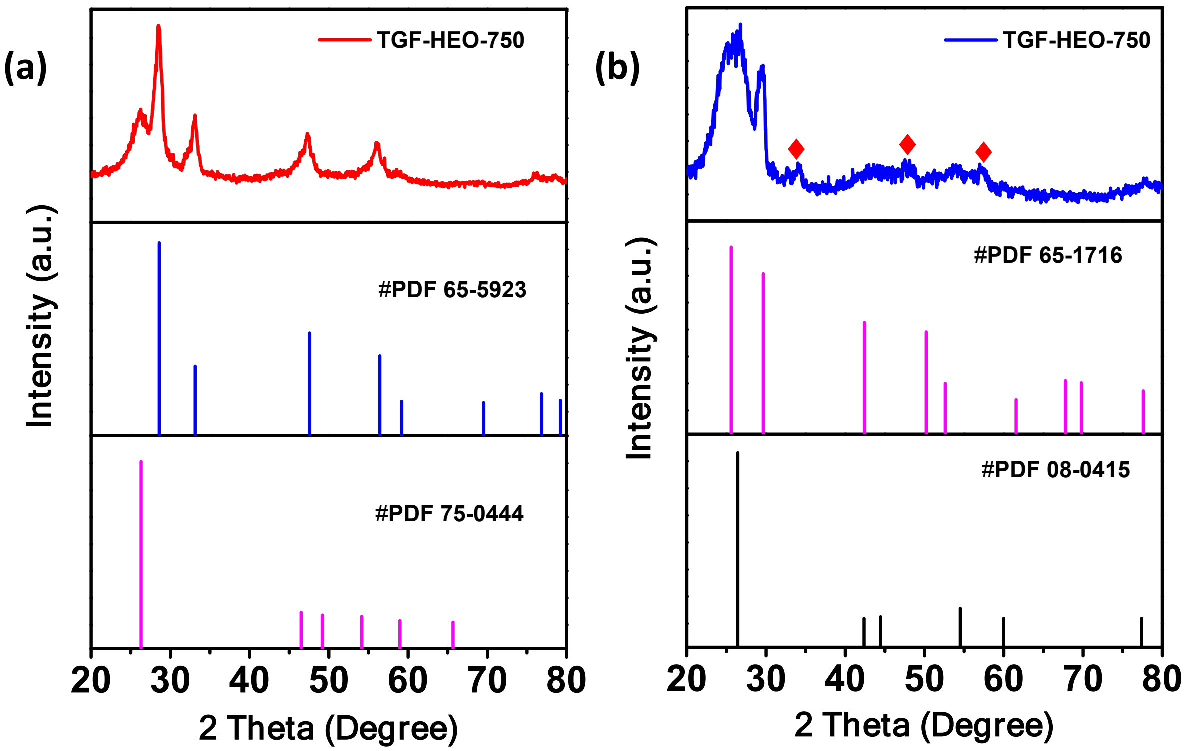

Figure S15. XRD pattern of TGF-HEO-750 (a) before and (b) after charge-discharge cycles.

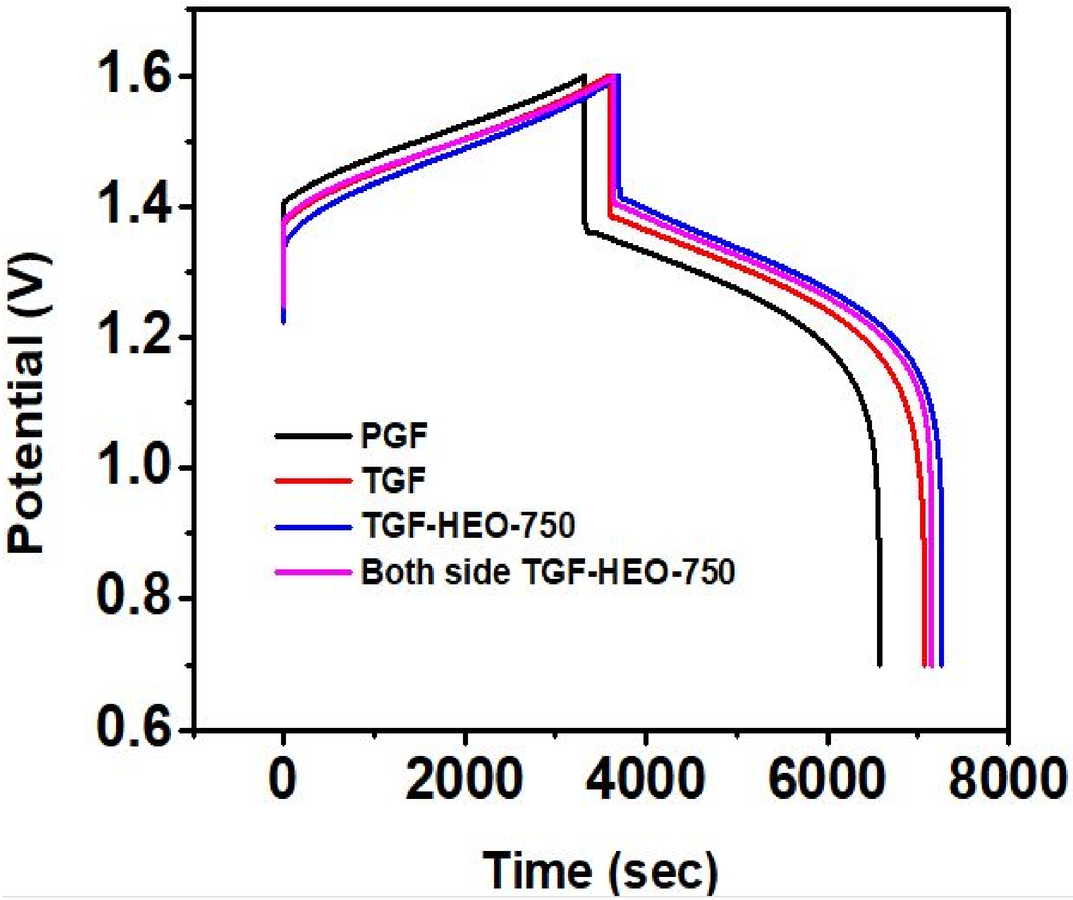

Figure S16. Charge-discharge profiles of the cell PGF, TGF, TGF-HEO-750, and both sides TGF-HEO-750 at the current density of 80 mA cm^{-2}.

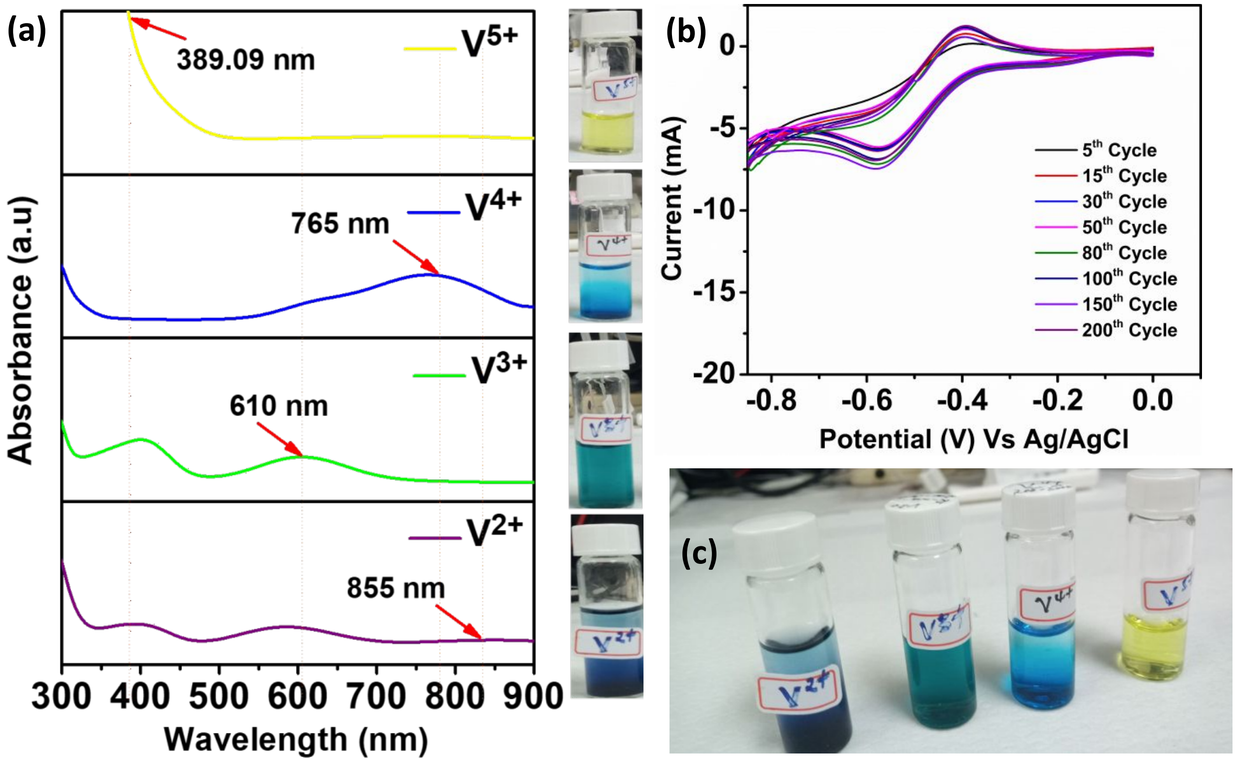

Figure S17. (a) UV-Vis spectra of various vanadium electrolytes (1.6 M $VOSO_4$ + 4.6 M H_2SO_4 solution with DI water by 20 times dilution). (b) Repeated test at 10 mV s^{-1} scan rate. (c) Vanadium electrolyte color.

Table S5 Comparison of the CE, VE, and EE of the TGF-HEO-750 material with those of previously reported metal and metal oxide-based materials.

Materials	Electrolyte concentration	Membrane type	Current density (mA cm^{-2})	CE (%)	VE (%)	EE (%)	Ref
TGF-HEO-750	1.6 M $VOSO_4$ + 4.6 M H_2SO_4	Nafion 212	120	97.77	81.42	79.61	**This work**
			160	98.10	74.76	73.34	
$BiVO_4$-GF	1.6 M $VOSO_4$ + 4.6 M H_2SO_4	Nafion 212	100	97.23	75.42	77.57	[4]
3D annealed WO_3 NWs/GS- GF	1.6 M $VOSO_4$ + 2.5 M H_2SO_4	Nafion 117	80	94.98	83.69	79.49	[5]
TiC-GF	1.6 M $VOSO_4$ + 4 M H_2SO_4	Fumasep® FAP 450	100	98.63	73.60	74.62	[6]
Bi_2O_3/CNTs-GF	1.5 M $VOSO_4$ + 3 M H_2SO_4	Nepem115	50	94.78	83.56	88.15	[7]
Ti/IrO_2:Ta_2O_5	1.7 M $VOSO_4$ + 4 M H_2SO_4	Nafion 117	40	90	90	81.0	[8]
Nb-doped h-WO_3 NWs/GF	1.6 M $VOSO_4$ + 2.5 M H_2SO_4	Nafion® 117	80	93.16	83.83	78.10	[9]
Mn_3O_4/CF	2 M $VOSO_4$ + 2.5 M H_2SO_4	Not specify	40	83.5	91.0	76.0	[10]
Ir/CF	0.5 M $VOSO_4$ + 2 M H_2SO_4	Nafion117 ®	20	79.7	87.5	69.7	[11]
$W_{18}O_{49}$ NWs /GF	1.6 M $VOSO_4$ + 3 M H_2SO_4	Nafion 117	80	94.2	85.0	80.1	[12]
CeO_2/GF	2 M $VOSO_4$ + 2 M H_2SO_4	Nafion 117	100	87.9	84.2	74.0	[13]
Ta_2O_5-GF	1.6 M $VOSO_4$ + 2.5 M H_2SO_4	Nafion 117	80	94.8	78.1	73.7	[14]

References

(1) Kabtamu, D. M.; Chen, J.-Y.; Chang, Y.-C.; Wang, C.-H. Water-activated graphite felt as a high-performance electrode for vanadium redox flow batteries. *Journal of Power Sources* **2017**, *341*, 270-279. DOI: 10.1016/j.jpowsour.2016.12.004.

(2) Sankar, A.; Michos, I.; Dutta, I.; Dong, J.; Angelopoulos, A. P. Enhanced vanadium redox flow battery performance using graphene nanoplatelets to decorate carbon electrodes. *Journal of Power Sources* **2018**, *387*, 91-100.

(3) Tegegne, B. G.; Kabtamu, D. M.; Li, Y.-Z.; Ou, Y.-T.; Huang, Z.-J.; Hsu, N.-Y.; Ku, H.-H.; Wang, Y.-M.; Wang, C.-H. N-methylphenothiazine as stable and low-cost catholyte for nonaqueous organic redox flow battery. *Journal of Energy Storage* **2023**, *61*, 106753.

(4) Kabtamu, D. M.; Li, Y.-Z.; Bayeh, A. W.; Ou, Y.-T.; Huang, Z.-J.; Chiang, T.-C.; Huang, H.-C.; Wang, C.-H. Bivo4-Decorated Graphite Felt as Highly Efficient Negative Electrode for All Vanadium Redox Flow Batteries. *Available at SSRN 4112168*.

(5) Kabtamu, D. M.; Chang, Y.-C.; Lin, G.-Y.; Bayeh, A. W.; Chen, J.-Y.; Wondimu, T. H.; Wang, C.-H. Three-dimensional annealed WO 3 nanowire/graphene foam as an electrocatalytic material for all vanadium redox flow batteries. *Sustainable Energy & Fuels* **2017**, *1* (10), 2091-2100.

(6) Ghimire, P. C.; Schweiss, R.; Scherer, G. G.; Wai, N.; Lim, T. M.; Bhattarai, A.; Nguyen, T. D.; Yan, Q. Titanium carbide-decorated graphite felt as high performance negative electrode in vanadium redox flow batteries. *Journal of Materials Chemistry A* **2018**, *6* (15), 6625-6632.

(7) Chu, Y.; Zhou, H.; Zhao, H. Bismuth trioxide modified carbon nanotubes as negative electrode catalysts for all vanadium redox flow batteries. *Int. J. Electrochem. Sci* **2020**, *15*, 7733-7743.

(8) Raghu, S. C.; Ulaganathan, M.; Lim, T. M.; Kazacos, M. S. Electrochemical behaviour of titanium/iridium (IV) oxide: Tantalum pentoxide and graphite for application in vanadium redox flow battery. *Journal of power sources* **2013**, *238*, 103-108.

(9) Kabtamu, D. M.; Chen, J.-Y.; Chang, Y.-C.; Wang, C.-H. Electrocatalytic activity of Nb-doped hexagonal WO 3 nanowire-modified graphite felt as a positive electrode for vanadium redox flow batteries. *Journal of Materials Chemistry A* **2016**, *4* (29), 11472-11480.

(10) Kim, K. J.; Park, M.-S.; Kim, J.-H.; Hwang, U.; Lee, N. J.; Jeong, G.; Kim, Y.-J. Novel catalytic effects of Mn 3 O 4 for all vanadium redox flow batteries. *Chemical Communications* **2012**, *48* (44), 5455-5457.

(11) Wang, W.; Wang, X. Investigation of Ir-modified carbon felt as the positive electrode of an all-vanadium redox flow battery. *Electrochimica Acta* **2007**, *52* (24), 6755-6762.

(12) Bayeh, A. W.; Kabtamu, D. M.; Chang, Y.-C.; Chen, G.-C.; Chen, H.-Y.; Liu, T.-R.; Wondimu, T. H.; Wang, K.-C.; Wang, C.-H. Hydrogen-Treated Defect-Rich W18O49 Nanowire-Modified Graphite Felt as High-Performance Electrode for Vanadium Redox Flow Battery. *ACS Applied Energy Materials* **2019**, *2* (4), 2541-2551. DOI: https://doi.org/10.1021/acsaem.8b02158.

(13) Bayeh, A. W.; Lin, G.-Y.; Chang, Y.-C.; Kabtamu, D. M.; Chen, G.-C.; Chen, H.-Y.; Wang, K.-C.; Wang, Y.-M.; Chiang, T.-C.; Huang, H.-C. Oxygen-vacancy-rich cubic CeO2 nanowires as catalysts for vanadium redox flow batteries. *ACS Sustainable Chemistry & Engineering* **2020**, *8* (45), 16757-16765.

(14) Bayeh, A. W.; Kabtamu, D. M.; Chang, Y.-C.; Chen, G.-C.; Chen, H.-Y.; Lin, G.-Y.; Liu, T.-R.; Wondimu, T. H.; Wang, K.-C.; Wang, C.-H. Ta2O5-Nanoparticle-Modified Graphite Felt As a High-Performance Electrode for a Vanadium Redox Flow Battery. *ACS Sustainable Chemistry & Engineering* **2018**, *6* (3), 3019-3028. DOI: 10.1021/acssuschemeng.7b02752.
